# Evaluating online nutrition information: a scoping review of young adults’ source preferences and criteria for credibility and trustworthiness

**DOI:** 10.3389/fdgth.2026.1784563

**Published:** 2026-06-26

**Authors:** Cassandra A. Omane, Sarah Forberger

**Affiliations:** 1 Department for Prevention and Evaluation, Leibniz-Institute for Prevention Research and Epidemiology – BIPS, Bremen, Germany; 2 Leibniz Science Campus Digital Public Health, Bremen, Germany; 3 Department of Health Science, University of York, York, United Kingdom

**Keywords:** credibility, digital health, health information, healthy diet, information sources, nutrition information, trust, young adult

## Abstract

**Introduction:**

The use of the internet to search for different kinds of information has become part of everyday life also for nutrition information. Therefore, it is important that the information is evidence-based, credible, and trustworthy. Assessing this is complicated by the declining importance of traditional health information gatekeepers and the rapid flow of information. This scoping review examines factors that influence the trustworthiness and credibility of digital information sources on healthy diets from the perspective of young adults.

**Methods:**

We conducted a scoping review with a comprehensive search of 5 databases and grey literature up to 12/2024, following the PRISMA-ScR guidelines. Two reviewers performed a two-stage screening process. The coding of trust- and credibility-influencing factors is based on the Misinformation Receptivity Framework and the Online Credibility Framework.

**Results:**

48 papers were included, identifying 82 factors concerning the formation of trust and credibility in nutrition information. The most frequently reported factors are clarity of the information (*n* = 14), prior background knowledge (*n* = 13), and receiver's education (*n* = 7). A small number of the included papers report on medium-, context-, or design-related factors (*n* = 23, *n* = 12, *n* = 8), such as the expertise of the information author, the communication context, or the surface attractiveness of a digital platform. 46 additional factors not covered by the two frameworks were identified. 75% of the papers do not report the study participants’ ethnicity (*n* = 36). 25% of the papers included a multi-ethnic sample (*n* = 12).

**Discussion:**

Our findings suggest that most studies focus primarily on the receiver's characteristics. The existing literature often neglects factors like the author's reputation, the usability of digital platforms, and the accessibility of information. In addition, other studies have found that ethnicity and nationality can strongly influence assessments of information trustworthiness and credibility. However, this is not systematically reported in the analyzed papers. This shows that further research is needed on trust- and credibility-inducing factors. Furthermore, the results underscore the need for research that incorporates diverse perspectives and cultural backgrounds to achieve a comprehensive understanding of this critical area.

**Registration:**

This scoping review has been pre-registered at OSF under DOI 10.17605/OSF.IO/5QPV6.

## 1 Introduction

The use of the internet has become part of everyday life (1). This also applies to young people. “Almost 80 percent of people aged between 15 and 24 used the internet” daily in 2023, according to the International Telecommunication Union (ITU), the United Nations specialized agency for information and communication technologies (2). In 2024, 97% of people aged 16 to 29 living in the EU reported daily Internet use (3).

This trend shapes where and how information is provided, searched, and accessed, extending to more specific information, such as health information (4). Several studies suggest that 61.5% - 78% of young adults in non-clinical samples search for health information on the internet at least weekly (5–9). However, factors other than the searcher's age also influence the search for health information. Several studies indicate that gender strongly influences how health and nutrition information is searched, processed, and utilized (10–14).

Furthermore, research has consistently found that an individual's health status and previous disease experiences notably influence how individuals seek health and nutrition information. A diagnosis may shape the focus of the information search itself (15), while prior exposure to a disease can influence how individuals perceive their risk of developing further health problems (16). As a result, personal experience with a disease can strengthen motivation to actively seek health information.

In general, health information is defined as “information relating in particular to general knowledge about health, diseases, their effects and their progression; measures to maintain health (prevention and health promotion); early detection, diagnosis, treatment, palliation, rehabilitation and aftercare of diseases and related medical decisions; care and coping with illness and everyday life with an illness” (17). In general, health information has the aim of “empowering people in making health-related decisions” and “to enable them to organize their everyday lives from a health perspective” (18). Health information can enable people to take responsibility for their health and to play an active role in maintaining their health and “therefore informing people [..] is of utmost importance” (19–25). It empowers individuals to manage their health effectively and to adopt behaviors that promote well-being, such as a healthy diet.

A healthy diet plays an important role in terms of overall health (26, 27). This is particularly evident in the association between the consumption of high-caloric foods and non-communicable diseases, including obesity, hypertension, and inflammation (27–29), which in turn are associated with higher morbidity and mortality (30). Excessive consumption of sugar, salt, and highly processed foods is widely regarded as detrimental to health. In contrast, a healthy diet, defined as a pattern of food intake that has beneficial effects on health (28), is typically characterized by the intake of fresh fruits, vegetables, nuts, whole grains, and less-processed food options (27–29, 31).

Given the assumption that unhealthy foods are particularly popular among young adults due to their relatively low cost, strong marketing appeal, and widespread availability (32), it is necessary to explicitly examine young adults’ information behavior regarding healthy diets.

Young adulthood is a developmental phase during which values, norms, and habits are not yet as firmly established. This makes it a possible key window of opportunity to shape long-term nutrition behavior. Contributing factors include the increased average age at leaving the parental “nest” due to rising housing costs, the delay in childbirth and parenthood compared with previous generations, a prolonged phase of post-secondary and tertiary education, and a postponed labor market entry (33). This extended window of opportunity to influence young adults’ behavior can be used to lay the foundation for healthy behaviors. In addition, various studies argue that human brain development continues into the mid- to late twenties (32, 34, 35), affecting skills such as planning, impulse control, and decision-making (32, 34, 35). Therefore, recent research findings argue that classical markers of adulthood have shifted, leading towards a prolonged status as a young adult (36). Consequently, this scoping review expands the operational definition of “young adult” to 18-29 years.

Many young adults are interested in specific information about healthy diets. Statistics show that between 70% and 92% of young adults use the internet to search for health and nutrition information (37–40). The most searched topics were healthy diets and nutrition in general, weight loss, exercise/fitness, and specific recipes or diet plans (41–44). Young adults use different starting points to search for health information in digital environments, such as general search engines (e.g., Google) and social media (45). Further, different platforms are used for different purposes (46). While Facebook is described as a “boredom killer,” and engagement with health content occurs more by chance through long scrolling, similar to TikTok, YouTube is purposely used to search for health content published by health professionals (46, 47). Moreover, Instagram is described as “*a source of inspiration for health and wellness*,” and Buzzfeed's “Tasty Channel” is utilized for “*easy to follow recipe videos and tutorials*” (46).

This thirst for knowledge is met with an information landscape that has changed significantly in recent years (48). Although more and more health organizations have increased their efforts to provide publicly available health information (25), the relationship between health professionals and laypersons is still characterized by information asymmetries. This leads to a high level of uncertainty among laypeople (49). The unequal relationship between the two groups is now facing additional challenges: Digital information sources are gaining importance due to their instant (remote and multiple) access to information, the ability to store large volumes of data, and faster information addition (50). The ease and speed with which information can now be published online has led to much information that is not subject to traditional quality control or does not adhere to predefined writing and review processes (51). Classical gatekeepers of information (e.g., healthcare professionals or established organizations) lose their interpretive sovereignty (48, 52). Therefore, the expectations regarding individuals’ digital health literacy are high and increasing. These include having a clear sense of the credibility of multiple sources, being familiar with different message categories, and understanding the various layers of online dissemination of different content types (51). Internet users are largely left to their own judgment when assessing the credibility and trustworthiness of information sources (51, 53).

Issues of trust and credibility have a long-standing research tradition, having been explored across various disciplines (e.g., information science, sociology, psychology, economics, political science). Consequently, there are many different definitions of both concepts depending on the context (54, 55).

Valentini defines credibility as “the quality of being believed or accepted as true, real, or honest” (56). The authors argue that credibility should not be underestimated, as it is a decisive prerequisite for building trust in decision-making (55–57). Trust is defined as “a positive belief about the perceived reliability of, dependability of, and confidence in a person, object, or process” (58). Trust is an important determinant of behavior and develops over time through interactions among different parties (59–61). Several definitions indicate that both concepts have been historically closely linked and are continuously defined as part of each other (55, 57, 62–66). However, based on the review by Sbaffi and Rowley (66) of trust in web-based health information, there is ongoing discussion about the precise distinction between trust and credibility, as well as their relationship. Therefore, this review will incorporate both concepts.

This scoping review explicitly addresses trust and credibility concerning online health information focused on a healthy diet and synthesizes trust- and credibility-building factors among the target group of 18- to 29-year-olds. Prior reviews analyzed research findings to provide an overview of trust cues that consumers use to assess food quality and safety (67) or focused on the interplay among demographic characteristics, web-based health information, and assessments of credibility and trust (66). Other studies, in turn, investigated other age groups (13–18-year-olds) and their interaction with online health information and trust-building processes (68) or compared how the cultural background of participants influences trust and credibility assessments of online health information (69) or differences in general trust in healthcare systems between different countries (70).

As such, this scoping review aims to examine:

1. Which digital information sources for a healthy diet do young adults use?
2. What factors contribute to young adults considering digital sources of information on healthy diets to be trustworthy or credible?

## 2 Methods

This scoping review follows the methodology proposed by Arksey & O’Malley (67) and the PRISMA-ScR-Checklist (PRISMA Extension for Scoping Reviews) (71) (Supplementary File 1).

### 2.1 Study objectives

The scoping review adheres to the PCC (Population, Concept, Context) framework (72, 73) to define the review questions Table 1.

**Table 1:** Dimensions of the PCC framework.

Framework category	Category title	Definitions
Population	Young adults	Individuals in the age range of 18 to 29 years old.
Concept	Credibility of information	Credibility refers to “judgments made by a perceiver concerning the believability” (142) and it is “frequently attached to objects of assessment” (55). It can be differentiated into different types: source, media and message credibility (55, 57) Source credibility refers to the personal characteristics of a communicator which “hinder or enhance specific communication efforts” (56). Media credibility measures the extent to which the public views a specific communication channel or medium as a reliable source of information (56). Message credibility is of particular importance in circumstances where the recipient is not familiar with the communicator. In such cases, the recipient must consider the characteristics of the message, including its content, structure and delivery (53, 56).
	Trust in information	Trust is defined as “an expectancy of positive (or nonnegative) outcomes that one can receive based on the expected action of another party in an interaction characterized by uncertainty” (143).
Context	Digital information sources for a healthy diet	Digital information sources include, but are not limited to:
		• online search engines
		• blogs
		• online communities/social networks
		• online appearances of organizations, online consultations of health/nutrition professionals
		• online advertisement of food industry (144–148)

Various studies suggest that the assessment of the trust and credibility of information is heavily influenced by demographic factors, such as recipients’ age (66, 74–76). Therefore, the scoping review includes young people between 18 and 29 years.

Many young people at that age are moving out of their family homes for the first time. Therefore, they are no longer subject to their parents’ control regarding their eating and health behaviors (36). This phase is referred to as tertiary socialization or adult socialization. According to Fleischer and Hajok, “the individual constantly adapts in interaction with his social environment, further developing his values, attitudes and behaviors, whereby the fundamental orientations and approaches (including those relating to the media) acquired to date [..] are not completely discarded, but rather varied and expanded.” (77)

This phase presents an opportunity to influence this age group and help them establish long-lasting eating habits. This age group is still capable of influencing and modifying their behavior with relative ease, as it is not yet as firmly established (78). In light of the above, this age period presents a window of opportunity to adopt and consolidate healthy behaviors.

### 2.2 Protocol and registration

The protocol was published in advance on the Open Science Framework (OSF) (79).

### 2.3 Eligibility criteria

The inclusion and exclusion criteria were discussed beforehand and agreed upon by the research team. Detailed eligibility criteria are shown in Table 2.

**Table 2:** Eligibility criteria for the scoping review.

PCC dimension	Inclusion	Exclusion
*Participants*	studies that focus solemnly on young adults (18 to 29 years) or where results are reported for this specific age group	studies that do not include young adults as target group or participants (e. g. only adolescents, only elderly etc.)
	studies about young adults with or without pre-existing health conditions	Studies including young adults as parents but focusing on healthy nutrition for infants or children or focusing on nutrition during pregnancy
*Concept*	studies that ask their participants which digital information sources for healthy diets they use to inform themselves (only data on digital sources will be extracted)	intervention or feasibility studies that present information on healthy diets to the participants
	studies that identify trust-building aspects with regard to information about healthy diets	studies from the political sciences that focus on credibility and misinformation
	studies that identify factors for the credibility of online information about healthy diets	studies that focus on trust or credibility and other public health topics than healthy diets (e. g. trust in vaccines, trust in risk communication and food safety issues)
*Context*	studies that investigate only digital information sources for healthy diets as well as mixed studies which investigate analog and digital information sources	studies that focus only on analog information sources for healthy diets
*Additional criteria*	English or German	any other language (at full-text stage)
	full-text available	e. g. no full-text available
	research articles, grey literature article or report	books, dissertations and conference abstracts, other literature reviews (e. g. systematic or scoping reviews)
	all study designs	pre-defined lists of information sources to choose from as this would determine the results
	outcome: studies that only assess the participants’ knowledge of nutrition topics	Consumer information (e.g. advice on selecting or preparing food)

Recent research findings argue that classical markers of adulthood have shifted, leading towards a prolonged status as a young adult (36). Therefore, this scoping review deviates from the previously published protocol (79) by expanding the definition of “young adult” to 18-29 years.

### 2.4 Information sources

Five databases were searched until 10 December 2024: MEDLINE via PubMed, Web of Science Core Collection via Clarivate [SCI-EXPANDED, SSCI, AHCI, CPCI-S, CPCI-SSH, BKCI-S, BKCI-SSH, CCR-EXPANDED, IC], APA PsycINFO via Ovid, CINAHL via EBSCOhost, and AGRICOLA.

As the goal of this scoping review is to gain a comprehensive understanding of the information sources accessed and the trust- and credibility-spending factors, the decision to incorporate grey literature is based on several reasons.

Grey literature broadens the range of perspectives by incorporating voices and experiences that are sometimes underrepresented in academic circles, such as practitioners and community organizations. Many of these organizations are in direct contact with their target groups and therefore have a good understanding of their dietary habits and information-seeking behavior. Thus, the inclusion of grey literature enables the supplementation of existing scientific knowledge and a more realistic picture of young people's information practices.

### 2.5 Search

The electronic search strategy was developed and tested within the team in collaboration with an experienced librarian (LC). An iterative technique using the PRESS checklist (Peer Review of Electronic Search Strategies) was used to develop the search strategy (80). The search strategy was built around the following key terms: “trust,” “credibility,” “young adult,” “information,” and “healthy diet” and adapted for each included database. The search strategy for MEDLINE is reported in Supplementary File 2.

An extensive search for grey literature was conducted on the websites of both national and international organizations (Supplementary File 3), focusing on health and nutrition until 10 December 2024. This search utilized the following keywords: “healthy diet,” “trust,” “credibility”, and “information.”

### 2.6 Selection of sources of evidence

The resources found in the search were imported into the Rayyan software to conduct literature reviews. Deduplication, title/abstract screening, and full-text screening were performed in Rayyan. Two researchers screened titles, abstracts, and full-texts independently (SF & CAO). All conflicts were discussed, and a final decision was reached by consensus.

Grey literature was screened by a single researcher (CAO) to balance scientific rigor and efficiency. This approach was chosen because grey literature sources are heterogeneous, often including reports, policy documents, and organizational publications that are not subject to peer review. Screening by a single researcher enabled a pragmatic, resource-efficient process while maintaining transparency and methodological consistency. To ensure quality, the agreed upon inclusion and exclusion criteria (Table 2) were applied systematically, and any uncertainties were discussed with the research team and resolved through consensus. This method is consistent with established practices in scoping reviews (81, 82), in which a single reviewer screens grey literature to streamline the workflow without compromising the comprehensiveness of the review, provided that the process is clearly documented and justified.

**Figure 1:**
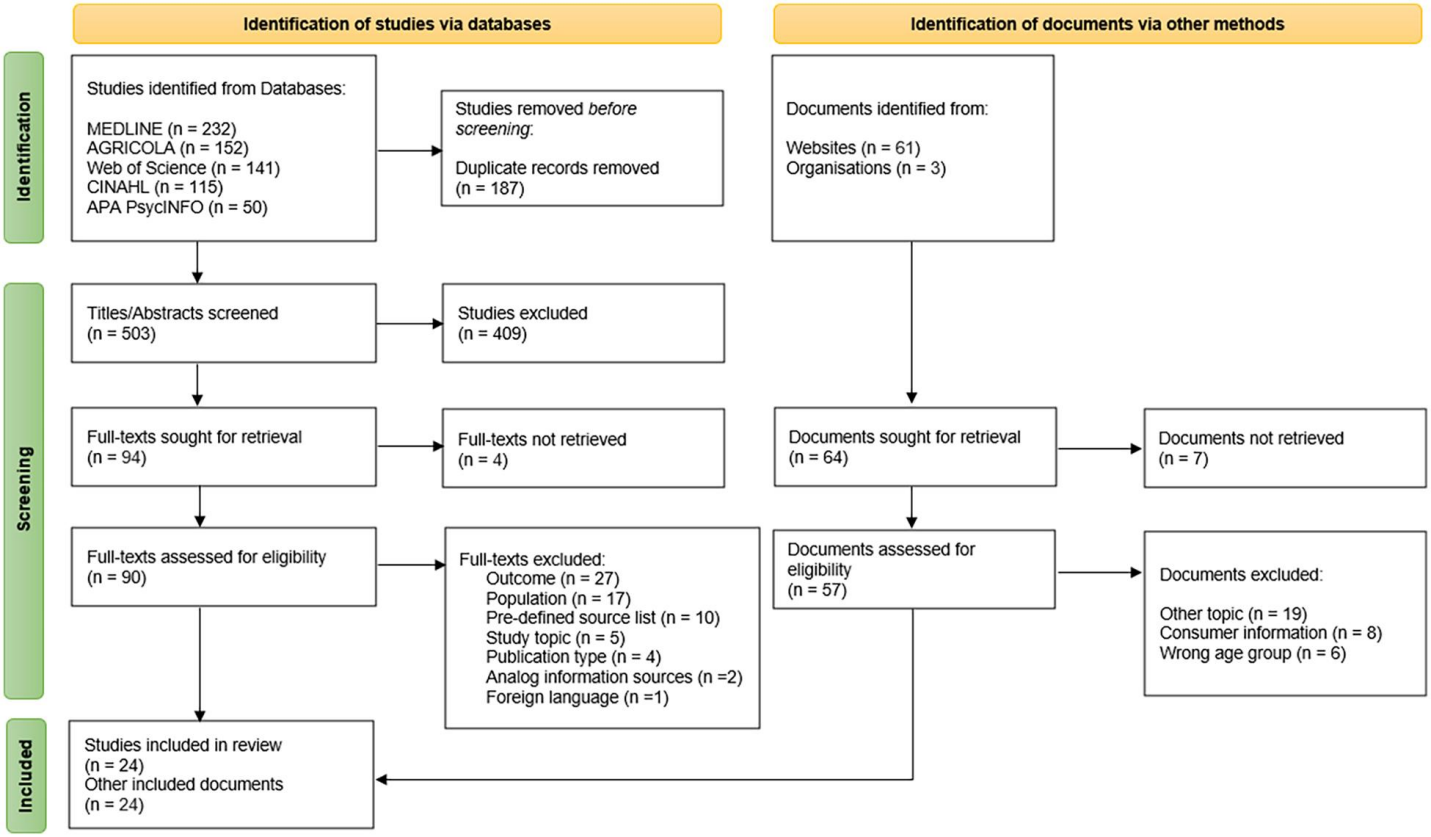
Prisma flow for this scoping review.

The complete PRISMA flowchart is reported within the results section of this article (Figure 1).

### 2.7 Data charting process and data items

Microsoft Excel was used to chart data from the included papers. One researcher (CAO) extracted data using a standardized, pretested form. […]

In addition to the metadata of the included papers (e.g., authors, publication date, etc.), two frameworks were utilized as a basis for the data extraction form: the *Misinformation Receptivity Framework* (MRF) (83) and the *Online Credibility Framework* (OCF) (84).

The model by Zmigrod et al. (83) is situated within political science and addresses the factors that contribute to the credibility of misinformation and to an individual's formation of trust in it. The model posits that when faced with new (mis)information, individuals weigh it against existing knowledge and assess its credibility and trustworthiness based on intrinsic and extrinsic factors. These factors, as outlined by Zmigrod et al. (83), influence an individual's perception of the information in question (83). Examples of these factors include prior beliefs and expectations, the discrepancy between old and new information, and personal views of the world and one's own identity (83). Furthermore, the framework by Zmigrod et al. (83) takes a psychological perspective on trust in newly received information and on how this information is weighted relative to already known information. Furthermore, this framework addresses susceptibility to false information and the factors that contribute to it (83).

As trust and credibility are strongly interconnected concepts and, in some cases, not clearly distinguishable from one another (55, 57), we included a second framework focusing on credibility. Wathen and Burkell (84) conducted an extensive literature review that resulted in a model exemplifying how users judge online credibility. The review (84) provides insights on “the general markers of credibility, and how different source, message and receiver characteristics affect people's perceptions of information” and the “assessment of credibility in the context of information presented on the Internet.”

One of the main findings of their review is that many credibility-influencing factors apply to both offline and online environments (84). Yet they also propose other factors that play a significant role, especially in the online context (84). Therefore, we combined both frameworks. Relevant passages from both frameworks were extracted and collected, with each passage forming the basis for a code.

A detailed list of the extracted data and their operationalization is provided in Table 3**.**

**Table 3:** Predefined categories for coding.

Domain/Area	Operationalization	
	Code	Explanation
Publication year	as stated in the publication	
Country and income level	Country: as indicated in the publication, if not indicated in the publication, country of first author affiliation	
	Income level: high-income, upper-middle, lower-middle, low-income	based on the World Bank country classifications by income level 2024–2025 (149)
Study type	quantitative, qualitative, mixed-methods	
Gender	gender distribution within the sample: only males, only females, mixed sample, mixed sample - female predominates mixed sample - male predominates	As soon as one gender accounted for more than 69% of the study participants within a sample, this was included in the coding and labeled with the code ‘mixed sample (… predominates)’.
Age	young adults (18–25) young adults and older (mean age < 25) young adults and older (mean age > 25)	studies include young adults, but also older participants: mean age of the participants is still <25 years studies include young adults, but also older participants: mean age of the participants is between 25 and 28 years
Nationality and ethnicity	as stated in the publication	If the ethnicity of the participants was not reported, only nationality has been coded.
Education (background and level)	university students (major not reported) university students (mixed majors) university students (health & nutrition) university students & individuals with degree nutrition experts	
Health status	not reported, healthy, diseased, mixed (healthy & diseased)	If the study did not state the health status, this was coded as ‘not reported’. The codes ‘healthy’ or ‘diseased’ were only assigned if specific statements were made in the article.
Access to information source	online, offline, mixed access (only & offline)	How the participants retrieved the searched information (e.g., online via social media, offline via friends or family)
Information sources	as stated in the publication	
Usage frequency of sources	most used source, 2nd most used, 3rd most used	in case the study reports on the most used information sources by the participants
Trust rating (most and least trusted sources)	most trusted, 2nd most trusted, 3rd most trusted least trusted, 2nd least trusted, 3rd least trusted	in case the study reports on the amount of trust which the participants place in specific information sources
Information receiver-related factors based on MRF & OCF (83, 84)	as stated in the publication e.g., includes but not limited to: beliefs or values of the receiver (MRF), “social location” of the receiver (OCF)	all characteristics of the information receiver that affect the extent to which they consider the information to be credible or trustworthy
Medium-/Source-related factors based on MRF & OCF (83, 84)	as stated in the publication e.g., includes but not limited to: expertise (OCF), reputation of the source (MRF)	all characteristics of the information medium or source itself that affect the extent to which the information is considered credible or trustworthy by third parties
Message-related factors based on MRF & OCF (83, 84)	as stated in the publication e.g., includes but not limited to: topic (OCF), framing (MRF)	all characteristics of the information or message itself that affect the extent to which the information is considered credible or trustworthy by third parties
Design-related factors based on OCF (83, 84)	as stated in the publication e.g., includes but not limited to: usability, surface/interface design, interactivity	all features of the design of the information that affect how credible or trustworthy the information is perceived to be by third parties
Context-related factors based on MRF & OCF (83, 84)	as stated in the publication e.g., includes but not limited to: systemic (in-)stability (MRF), time since message encountered (OCF)	all characteristics of the context in which the information is conveyed that affect the extent to which the information is considered credible or trustworthy by third parties

In cases where the frameworks did not cover an aspect, a new code was created and defined. An overview of all codes is provided in Supplementary File 4.

All included papers were first extracted and then coded by one researcher (CAO) by using the predefined coding template derived from the two frameworks presented in Table 3. The extraction and coding were subsequently verified by a second researcher (SF). Any discrepancies identified were marked, discussed, and resolved in consensus through revision of the coding.

### 2.8 Critical appraisal of individual sources of evidence

The purpose of a scoping review is to map the breadth and characteristics of the available literature. Therefore, no formal critical appraisal of the included sources was conducted.

### 2.9 Synthesis of results

The synthesis combined data charting with thematic coding.

Charting was used to map key characteristics of the included sources, including source type, country, study design, participant characteristics, and reported trust- or credibility-related factors.

Thematic coding followed a combined deductive and inductive approach. Initially, extracted passages were coded deductively using a predefined coding template based on the two conceptual frameworks presented in Table 3. Factors that did not fit the predefined categories were coded inductively and added as additional factors. Similar or overlapping codes were compared and merged where appropriate, and the resulting codes were grouped into broader cross-cutting themes, such as receiver-related, source-related, message-related, and medium-related factors. Peer-reviewed and grey literature sources were coded using the same procedure and integrated within the same synthesis matrix. Discrepancies were documented, discussed, and resolved by consensus.

Both data charting and coding were performed in Excel in order to document coding decisions, thematic groupings, and any changes to the coding resulting from internal team discussions as thoroughly as possible and to ensure transparency.

## 3 Results

### 3.1 Study selection

The database search identified a total of 690 articles. After excluding 187 duplicates, 503 articles remained for title and abstract screening. Following the exclusion of 409 articles and four articles that could not be retrieved, the full-text screening was conducted on 90 articles. In this stage, 66 articles were excluded for various reasons: publication type (*n* = 4), population (*n* = 17), incorrect outcome (*n* = 27), predefined source list (*n* = 10), foreign language (*n* = 1), analog information sources (*n* = 2), and study topic (*n* = 5). Ultimately, 24 studies were included for data extraction (Figure 1).

### 3.2 Grey literature selection

A total of 64 documents were identified through searches of relevant websites and organizations. Of these, 7 documents could not be retrieved, leaving 57 documents for assessment. 33 documents were excluded for various reasons: other topics (*n* = 19), incorrect age group (*n* = 6), consumer information (*n* = 8). Consequently, 24 documents were ultimately included for data extraction.

Therefore, in total, 48 papers (*n* = 24 scientific, *n* = 24 grey literature documents) were included. Detailed information on these papers is listed in Supplementary File 5.

### 3.3 General characteristics of included papers

**Figure 2:**
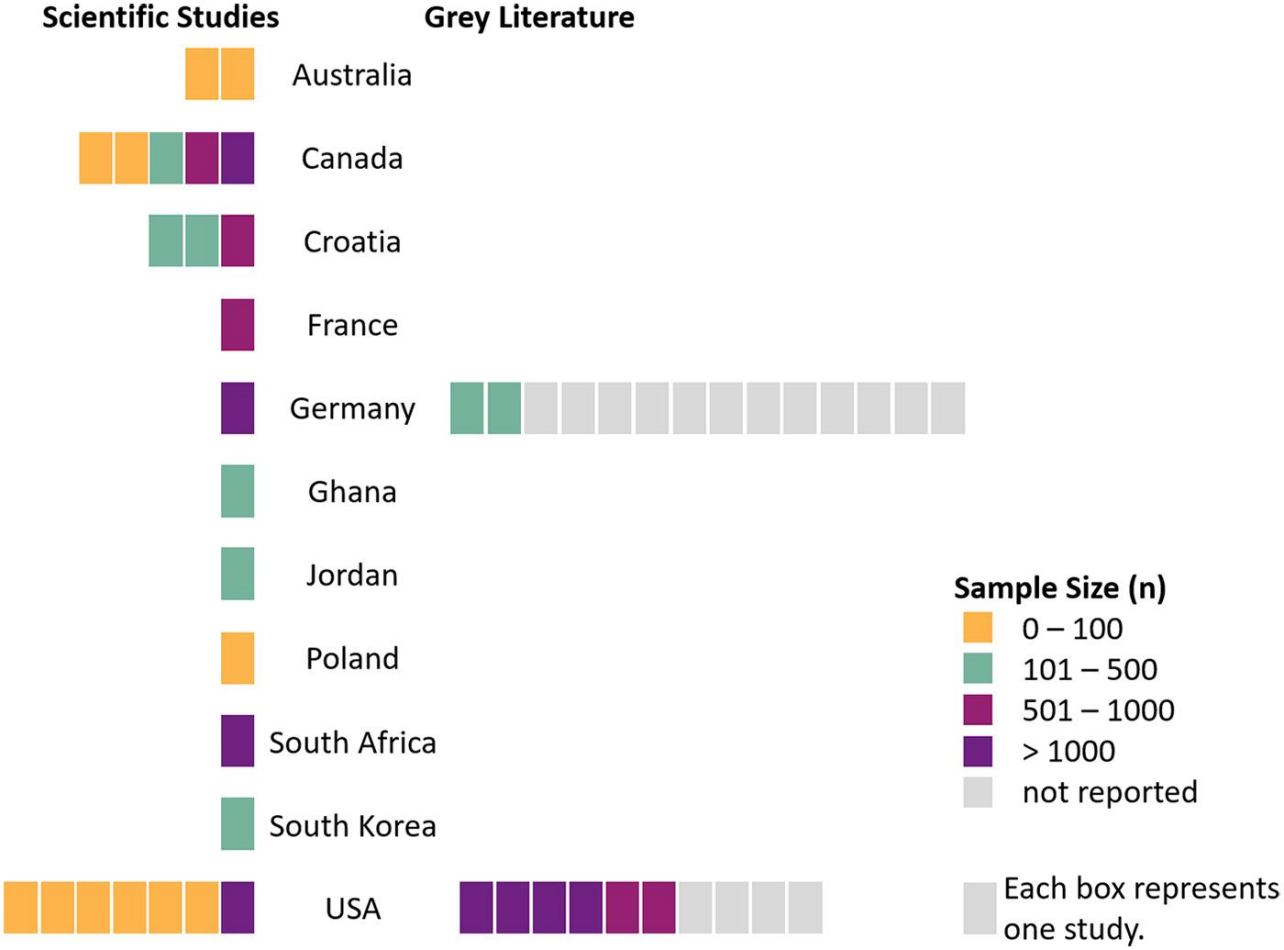
Countries and sample sizes of included studies.

Most of the included scientific studies cover high-income countries, particularly the United States (*n* = 7), Canada (*n* = 5), and Croatia (*n* = 3). The grey literature documents cover Germany (*n* = 14) and the United States (*n* = 10) (Figure 2).

The sample sizes ranged from 8 to 100 participants for 22.9% of the papers (*n* = 11) (Figure 2). A further 16.7% included 101-500 participants (*n* = 8). Additionally, 12.5% had sample sizes between 501 and 1000 participants (*n* = 6), while 14.6% had samples exceeding 1000 participants (*n* = 7). 33% of the papers did not report on the sample size (*n* = 16).

Among these papers, 29% included young adults aged 18 to 25 years (*n* = 14). 10% combined young adults with older participants, with a mean age under 25 years (*n* = 5), and 29% of the papers had a mean age between 25 and 29 years (*n* = 14). 31% did not report on the age of the participants (*n* = 15).

31% of the papers examined mixed samples comprising both female and male participants (*n* = 15). 17% had a predominantly female participant sample (*n* = 8). Two papers had exclusively male study samples (*n* = 2), one paper included only female participants (*n* = 1), 46% of the papers did not provide any information on participant gender (*n* = 22).

75% of the papers did not provide information on participants’ ethnicity (*n* = 36), while 25% included a multi-ethnic sample (*n* = 12).

44% of the papers did not report the educational background of the participants (*n* = 21), 40% recruited university students (*n* = 19), and 10% recruited both students and individuals with a degree (*n* = 5). A further 6% asked nutrition experts (*n* = 3).

Most papers did not disclose participants’ health status (*n* = 39). One paper investigated cancer survivors and their online search behaviors regarding nutrition health information (*n* = 1). One paper had a healthy participant sample (*n* = 1). The remaining seven papers included a mix of healthy individuals and participants with prior disease experience (*n* = 7).

#### 3.3.1 Topics searched by study participants

In 29% of the papers (*n* = 14), the study participants searched for general healthy diets, while 25% focused on healthy diets and how this information should be communicated to laypeople (*n* = 12). 8% (*n* = 4 each) of the papers dealt with healthy diet as part of general health behavior, or the use of apps or the internet to support a healthy diet or diets for athletes. 6% dealt with trending topics concerning healthy diets (e.g., personalized nutrition) (*n* = 3), and 8% focused on healthy dietary approaches or trust in communication about healthy diets and nutrition science (*n* = 4 each). Additionally, some papers addressed healthy diets in the context of specific diseases such as cancer, obesity, and osteoporosis (*n* = 1 each). At the same time, one study also addressed dietary supplements as part of a healthy diet (*n* = 1).

### 3.4 Types of information sources

The following Sections (3.4–3.6) describe the results by type of information source used. In general, information sources incorporate personal sources (such as health professionals, peers, family) and impersonal sources (e.g., health organizations, research institutes, food industry).

Sections 3.7–3.7.6 describe the various factors that can influence the trustworthiness and credibility of nutrition information. The operationalization of information sources, trustworthiness, credibility, and the various domains (receiver, information/message, medium/source, context, design) is presented in Tables 1, 3.

The most commonly reported source of information in the papers is the internet and websites in general (*n* = 27) (Table 4). In 37.5% of the papers, a mix of both online information and personal sources (e.g., friends/family, healthcare professionals) is reported (*n* = 18). Secondly, frequently reported are internet/website search, accompanied by asking/consulting healthcare professionals, nutritionists/dietitians, blogs, social networks/social media (*n* = 14). This is followed by asking friends/peers (in addition to online searches) and by watching YouTube or other online videos (each *n* = 12).

**Table 4:** Most cited information sources in the included papers overall.

Information source	overall papers (n)	grey literature (n)	studies (n)
Websites/internet in general	27	12	15
Healthcare professionals (e.g., physicians)	14	2	12
Nutritionists/dietitians	14	7	7
Blogs	14	8	6
Social networks/social media	14	9	5
Friends/peers	12	0	12
YouTube/Online videos	12	3	9

While Table 4 shows all information sources, Table 5 details the most-reported social media sources. The most commonly reported social media platforms for information are YouTube (*n* = 12), Facebook (*n* = 11), Instagram (*n* = 9), Twitter (*n* = 7), and TikTok (*n* = 6). Reddit (*n* = 2) and Pinterest (*n* = 1) were reported least frequently. Additionally, influencers/content creators were reported in 8 studies, but not explicitly linked to any one social media platform (*n* = 8).

**Table 5:** Most cited social media platforms as sources for nutrition information.

Information source	overall papers (n)	grey literature (n)	studies (n)
YouTube/online videos	12	3	9
Facebook	11	5	6
Instagram	9	3	6
Twitter	7	3	4
TikTok	6	1	5
Reddit	2	0	2
Pinterest	2	1	1

### 3.5 Most and least trusted information sources

In 65% of the papers (*n* = 31), no information was provided regarding the participants’ most trusted source. In three studies, online consulted healthcare professionals were identified as the most trusted source (*n* = 3). This also applies to online consulted dietitians and nutritionists (*n* = 3) or a combination of both, healthcare professionals and nutritionists accessed via online consultation (*n* = 3). Overall, there is no clear majority among the most-cited sources, as many are cited only once (e.g., medical journals, athletic trainers, research institutes).

83% of the papers did not report on the least trusted source (*n* = 40). Three papers reported social media (*n* = 3). Numerous other sources were referenced just once (e. g., TV or journal advertisements, someone with an online nutrition qualification, friends or peers) (*n* = 1 each), resulting in a lack of clarity on this topic as well.

### 3.6 Differences in scientific literature and grey literature regarding reported information sources

Differences become apparent in the frequency with which sources from grey literature or academic literature are reported (Table 6).

**Table 6:** Comparison of most cited information sources .

Frequency	Scientific studies	Grey literature
Most reported information source (n)	Website/Internet in general (15)	Website/Internet in general (12)
2nd most reported information source (n)	Friends/peers (12) Healthcare professionals (12)	Social media in general (9)
3rd most reported information source (n)	YouTube/Online videos (9)	Blogs (8)

The most frequently reported information source for grey literature and scientific studies is websites and the internet in general (*n* = 12, *n* = 15).

The second most frequently reported sources across both (scientific & grey literature) are personal information sources such as friends/peers (*n* = 12), healthcare professionals (*n* = 12), and social media in general (*n* = 9), in addition to online sources. A distinction arises in the third-most-frequently cited source. Scientific studies often report YouTube/Online videos (9), whereas in grey literature, blogs are reported (*n* = 8).

### 3.7 Factors influencing trust and credibility in digital nutrition information

Factors that contribute to trust in, or the credibility of, new information are categorized into five different domains: the receiver, the information or message, the medium or source, the context (in which the new information is encountered), and the design of the information (84).

In total across all analyzed papers, 82 factors have been reported. Of these 82 factors, 46 were not covered by the frameworks used (MRF & OCF) and were newly identified through the extraction and coding of relevant text passages.

The subsequent sections report on the trust- and credibility-related factors covered by the included studies and grey literature.

#### 3.7.1 Receiver-related factors about the formation of trust/credibility

**Figure 3:**
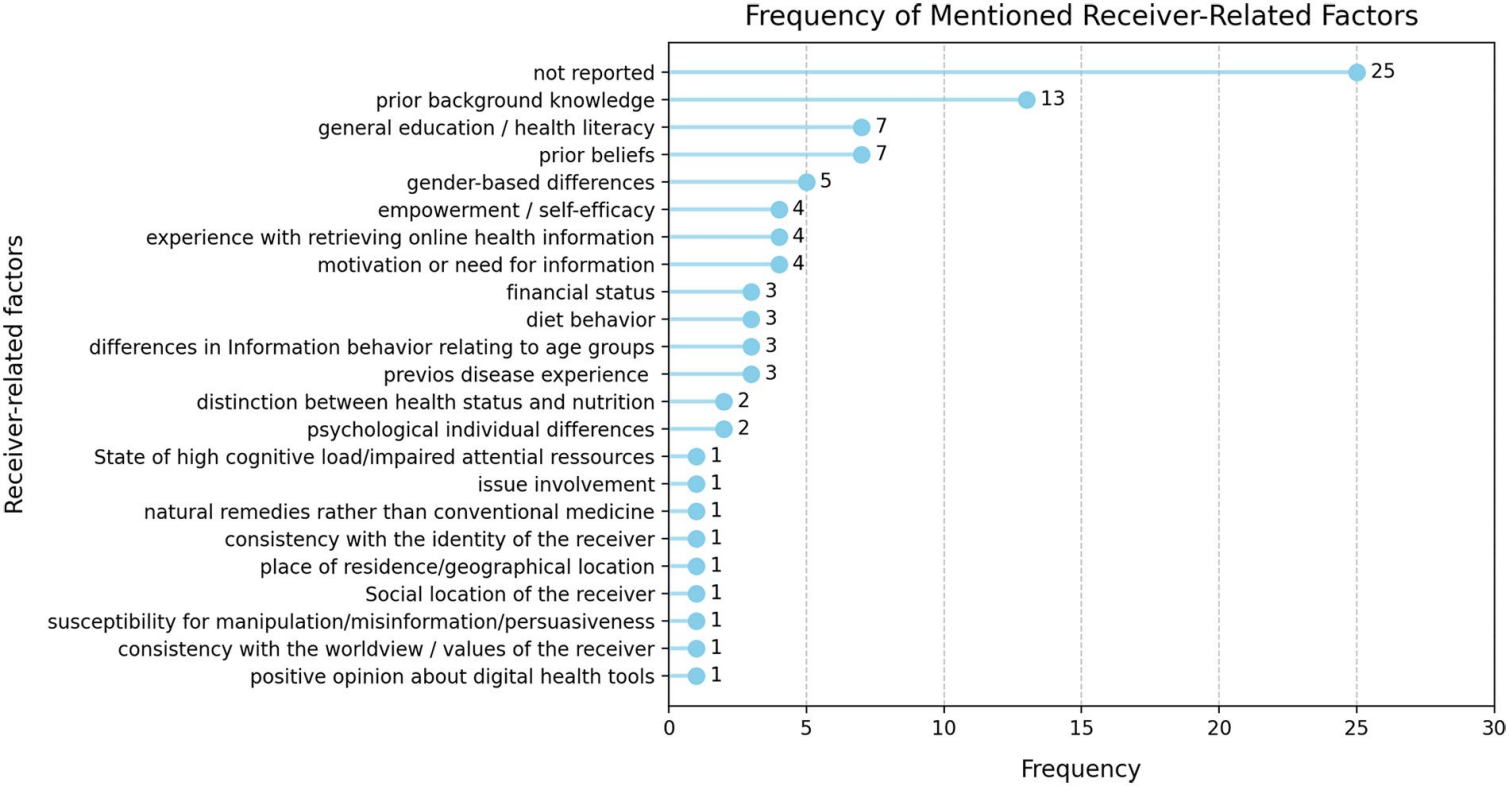
Frequency of all receiver-related factors in analyzed papers (studies and grey literature).

22 receiver-related factors were coded 69 times in 23 of the 48 analyzed papers. The most frequently reported receiver-related factors are presented in Figure 3. A comprehensive list of receiver-related factors and their explanations is provided in Supplementary File 4.

The most frequently reported factor is “prior background knowledge” of the information receiver (85–97) (*n* = 13). Second most frequently reported are “general education/health literacy” (85, 98–103) and “prior beliefs” of the individual (89, 91, 93, 97, 98, 100, 104) (each *n* = 7), while “gender-based differences” about the establishment of trust and credibility were reported by 5 papers (9, 98, 100–102) (*n* = 5). 52.1% of the papers did not discuss any receiver-related factors (*n* = 25).

Some recipient-related factors are reported with varying frequencies across website and social media content (Table 7). For example, the recipient's “prior background knowledge” is most frequently reported in relation to websites (*n* = 7), 4 times in relation to both websites and social media (*n* = 4), and 2 times in relation to social media content alone (*n* = 2). Other factors, such as “general education/health literacy” or “prior beliefs”, are reported with equal frequency in relation to website content and both (website and social media) (each *n* = 3).

**Table 7:** Three most reported factors across all areas: comparison website vs. social media.

Factors	Reported regarding website content (n)	Reported regarding social media content (n)	Reported regarding both (website & social media) (n)
**Receiver**			
prior background knowledge	7	2	4
general education/health literacy	3	1	3
prior beliefs	3	1	3
gender-based differences	2	0	3
**Information/message**			
clarity/ease of understanding/readability of information	7	4	3
personal relevance and/or individualized advice	7	3	1
practicability	4	2	1
support by data/examples/experts in the field	5	2	0
**Medium/source**			
expertise/knowledge of the source	2	4	5
official domain ending (.gov or.edu) or organizational affiliation of website	6	1	0
reputation of the source	3	1	0
authenticity of authors or content creators	0	4	0
likeability/goodwill of the source	1	3	0
**Context**			
amount of information as obstacle	4	3	0
communication context	4	2	0
traditional publishing process and/or fact-checking before publication	1	0	0
accessibility of information	1	0	0
echo chambers on social media (as space for inaccurate information)	0	1	0
**Design**			
surface attractiveness of digital platform	4	2	1
good quality of photos/videos	2	2	1
usability/accessibility	1	1	0
use of charts/infographics	1	1	0

The 12 newly identified receiver-related factors are detailed in Supplementary File 4. Those factors were not covered in one of the two frameworks (83, 84), but were reported in the analyzed papers. These factors include, among others: general education or health literacy of the information receiver (*n* = 7), experience with retrieving online information *n* = 4), and empowerment/self-efficacy through searching and finding information (*n* = 4).

Besides to that, 4 receiver-related factors were cited by the frameworks, but not covered by the analyzed papers in this review: relevance of the issue (OCF), ideological distance between new information and prior beliefs (MRF), general state of stress or relaxation (MRF) and stereotypes about the source or topic (OCF).

#### 3.7.2 Information- or message-related factors about the formation of trust/credibility

**Figure 4:**
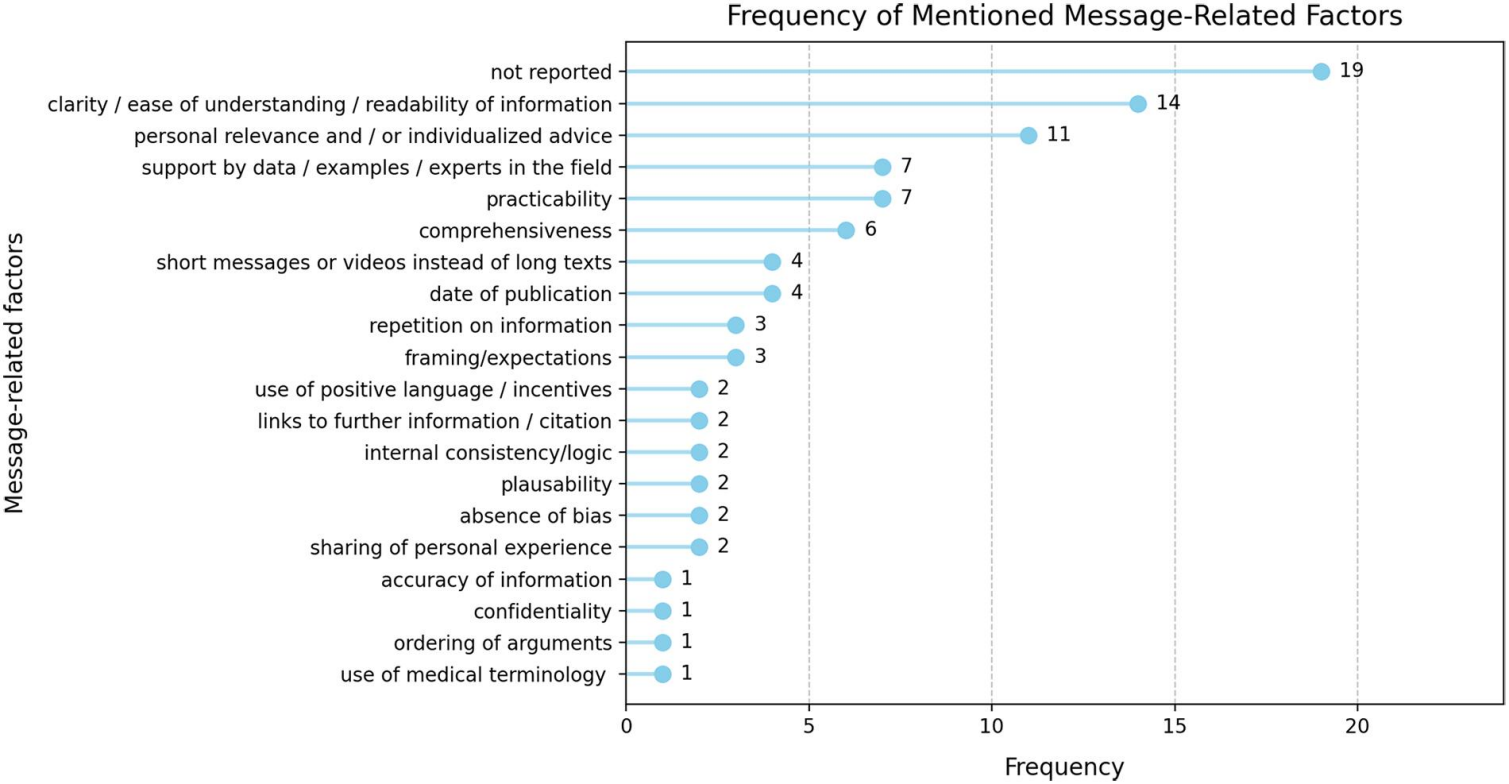
Frequency of all message-related factors in analyzed papers (studies and grey literature).

In total, 29 papers reported on message- or information related factors. 75 text passages about the message or information itself were coded (Figure 4). About one-third of the papers identified the “readability or clarity in word choice” of the message as the most frequently reported factor for establishing trust and credibility (9, 90, 92, 95, 98, 100, 105–112) (*n* = 14).

The second most frequently reported factor is “personal relevance/individualized advice” (9, 37, 86, 88, 91, 98, 102, 104, 111, 113, 114) (*n* = 11), while the “practicability” (93, 98, 102, 106, 107, 111, 115) and “support by data/examples/experts” of information (90, 94, 100, 104, 107, 115, 116) both rank third (each *n* = 7). More than a third of the papers did not discuss any message- or information-related factors (*n* = 19).

The “clarity of information”, its “personal relevance”, “practicability of information”, and the “support provided by data, examples, or experts” are all more frequently reported in relation to website content than to social media (*n* = 7 vs *n* = 4, *n* = 7 vs *n* = 3, *n* = 4 vs *n* = 2, *n* = 5 vs *n* = 2). In addition, the “clarity of information” is reported 3 times regarding both website and social media content (*n* = 3). At the same time, “personal relevance” and “practicability” are reported equally for website and social media content (each *n* = 1).

In total, 20 message- or information-related factors were reported, while one factor proposed by the OCF (“topic”) was not covered by the included papers. The analyzed papers cover 19 of the 20 message- or information-related factors.

The frameworks have not covered 13 factors concerning the information or message itself; the newly created codes for those factors are presented in Supplementary File 4. These factors include, among others: the clarity and readability of the information (*n* = 14), the personal relevance of the information (*n* = 11), and the practicability of the information for the receiver (*n* = 7).

#### 3.7.3 Medium- or source-related factors about the formation of trust/credibility

**Figure 5:**
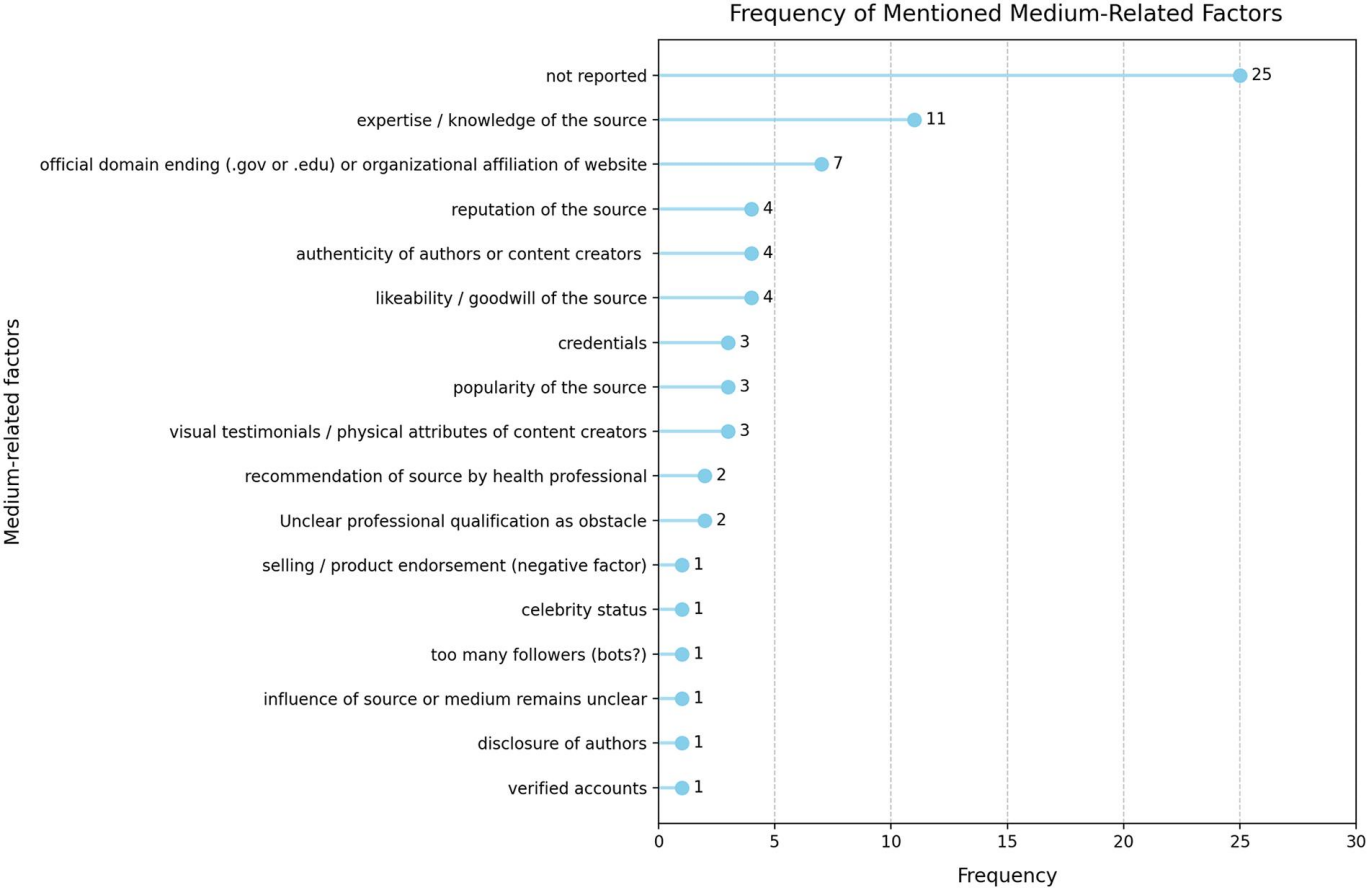
Frequency of all medium-related factors in analyzed papers (studies and grey literature).

16 medium or source-related factors were coded 51 times in 23 of the 48 papers (Figure 5). The most frequently reported factor is the “expertise or knowledge of the source” (37, 98, 100, 104, 105, 109, 112, 115, 117–119) (*n* = 11). An “official domain ending” such as “.gov” or “.edu” is reported second most frequently (37, 85, 89, 98, 101, 105, 118) (*n* = 7). The following factors were reported by 4 papers each: “reputation of the source”, “authenticity of authors or content creators”, and “likeability/goodwill of the source” (each *n* = 4). More than half of the papers did not report on medium- or source-related factors (*n* = 25).

An “official domain ending” is more often referred to websites than social media pages (*n* = 6 vs. *n* = 1) and the same applies to the reputation of the source (*n* = 3 vs *n* = 1) (Table 7). “Expertise or knowledge” is most frequently reported in the context of both social media content and websites (*n* = 5). Both the “authenticity of authors or content creators” and their “likeability” are reported less frequently in the context of websites than social media (*n* = 0 vs *n* = 4, *n* = 1 vs *n* = 3).

Supplementary File 4 details the 11 additional factors that were not included in the coding frameworks (MRF & OCF). These factors include, among others, an official domain ending such as “.org” or “.edu” (*n* = 7), the authenticity of authors or content creators (*n* = 4), and visual testimonials or physical attributes of content creators (*n* = 3).

#### 3.7.4 Context-related factors about the formation of trust/credibility

**Figure 6:**
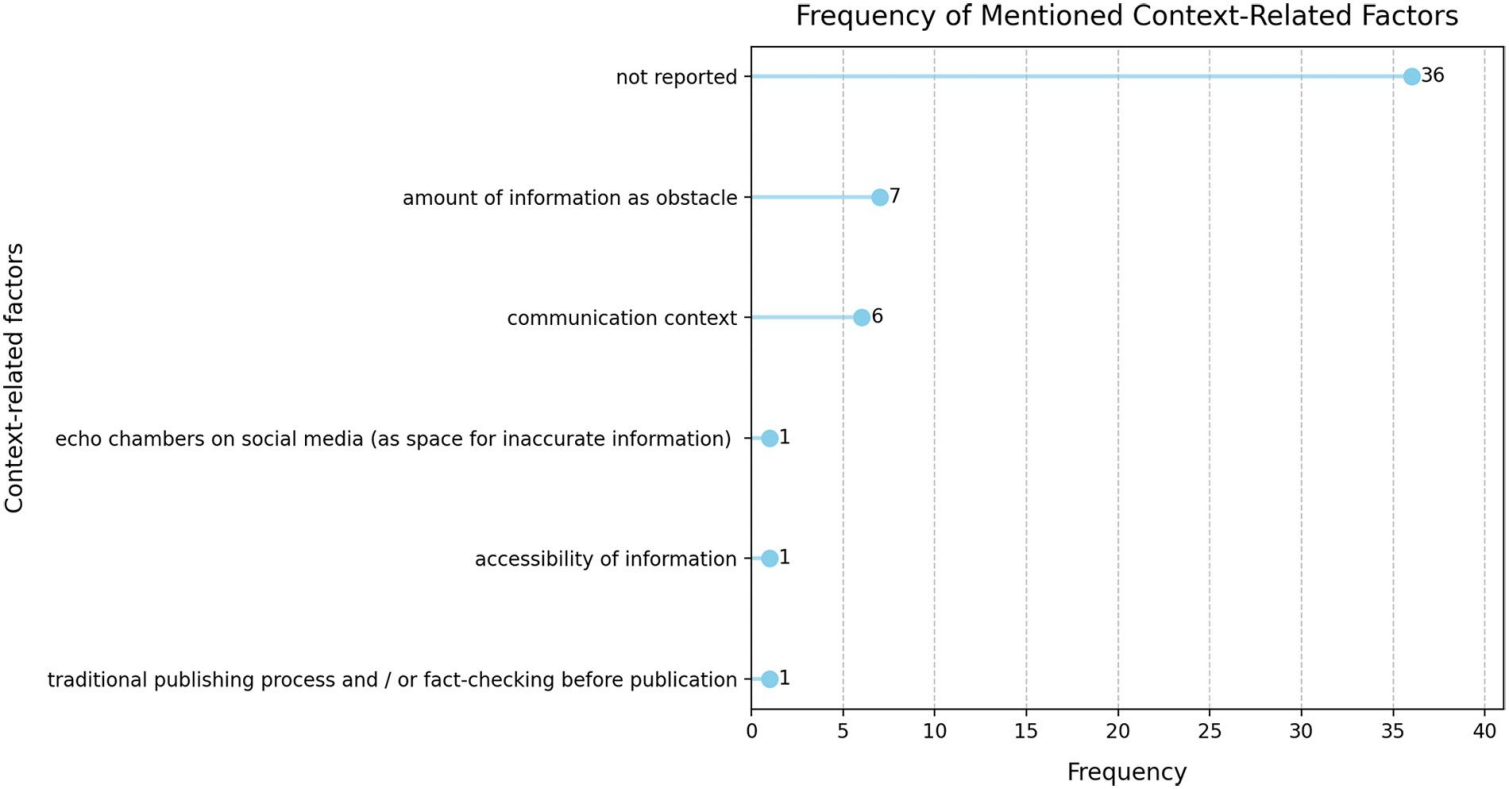
Frequency of all context-related factors in analyzed papers (studies and grey literature).

The most frequently reported context-related factors are presented in Figure 6. In total, 5 context-related factors were coded 16 times in 12 of the 48 papers. 3 factors proposed by the utilized frameworks were not covered by the included papers (relevance of noise to specific claim (MRF), time since contact with message (OCF), systemic (in-)stability (MRF)).

The most reported factor is the “amount of information as obstacle” (37, 92, 97, 108, 109, 120) for the establishment of trust or credibility (*n* = 7). The “communication context”, in which the new information is encountered, is reported second most frequently (90, 92, 102, 107, 110, 114) (*n* = 6). One paper each reported the “traditional publishing process and/or fact-checking before publication” (105), the “accessibility of information” (102), and “echo chambers on social media” (112) as interfering with trust and credibility of nutrition information (each *n* = 1). 36 of the papers did not report on context-related factors concerning the establishment of trust and credibility (*n* = 36).

Both the “amount of information” and the “communication context” are cited as factors influencing trust in relation to websites (each *n* = 4) (Table 7). These factors are reported less frequently in connection with social media content (*n* = 3, *n* = 2).

The factors not covered by either framework are presented in Supplementary File 4. These factors include: the sheer amount of information as an obstacle (*n* = 7), echo chambers on social media, the accessibility of information, and traditional publishing processes or fact-checking (each *n* = 1).

#### 3.7.5 Design-related factors with regard to the formation of trust/credibility

**Figure 7:**
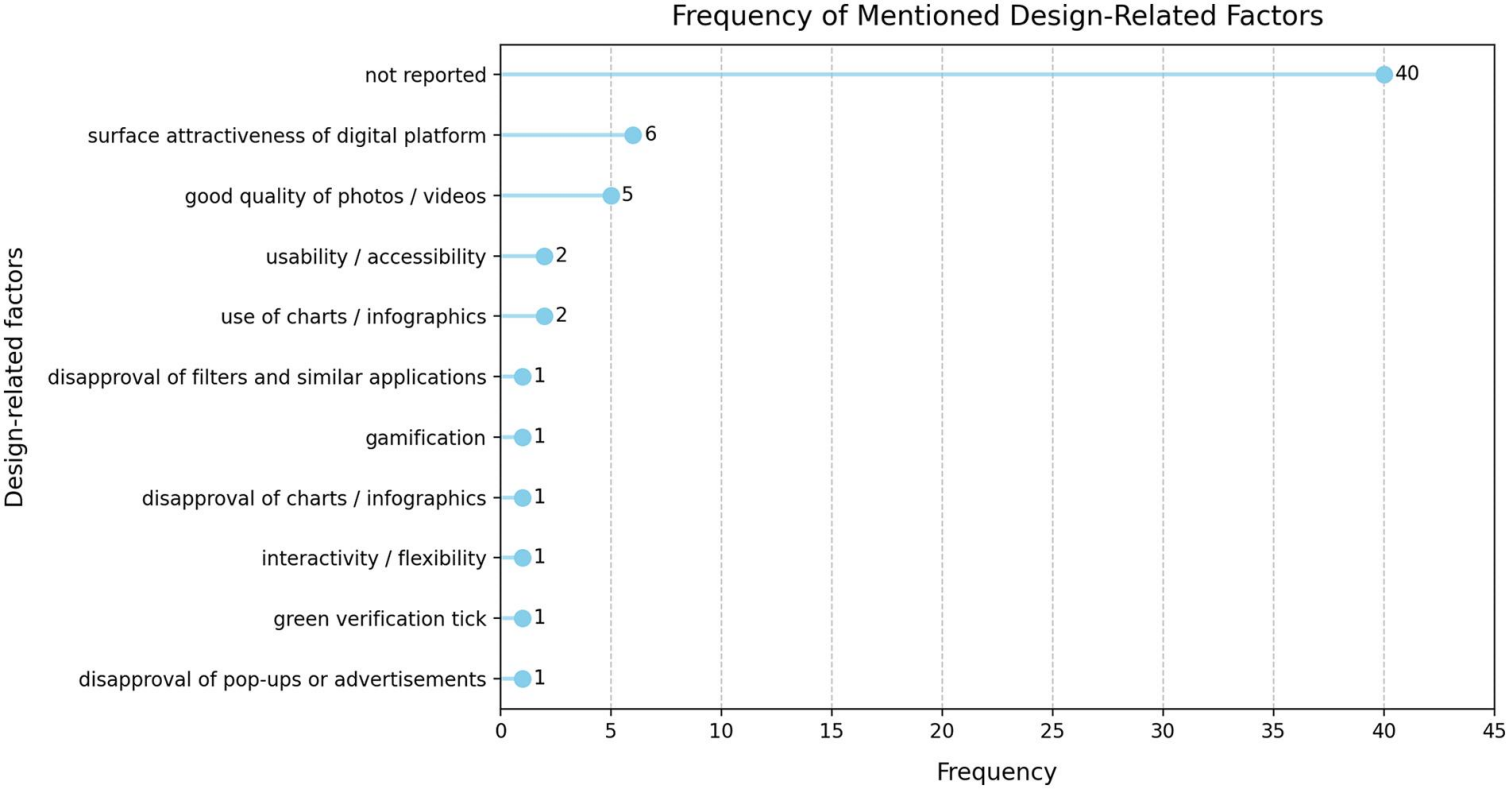
Frequency of all design-related factors in analyzed papers (studies and grey literature).

The most frequently reported design-related factors are presented in Figure 7. 8 of the 48 analyzed papers reported on 10 design-related factors. Two factors proposed by the OCF were not covered by the analyzed studies (speed of loading, design interface).

The “attractiveness of a website or application's surface” was identified as a significant factor in establishing trust (9, 85, 104, 105, 112, 121) (*n* = 6). An additional 5 papers reported on the “quality of photos or videos” as factor (9, 100, 102, 104, 112) (*n* = 5). Two papers each reported the “usability/accessibility” (9, 121) and the “use of charts/infographics” (100, 112) as relevant factors regarding the design (each *n* = 2). One paper each pointed to the use of charts or infographics on websites (85) and social media (112) as a trust- or credibility-inducing factor. The majority of papers (*n* = 40) did not discuss design-related factors.

The surface attractiveness of a digital platform is twice as often reported in relation to websites as it is in relation to social media content (*n* = 4 vs *n* = 2) (Table 7). All other design factors are reported with equal frequency for websites and social media content.

The 7 newly identified factors focusing on design-related aspects are detailed in Supplementary File 4. These factors include, among others: a good quality of photos or videos (*n* = 5) and the use or disapproval of charts or infographics (*n* = 3).

#### 3.7.6 Most frequently reported factors across all areas

**Figure 8:**
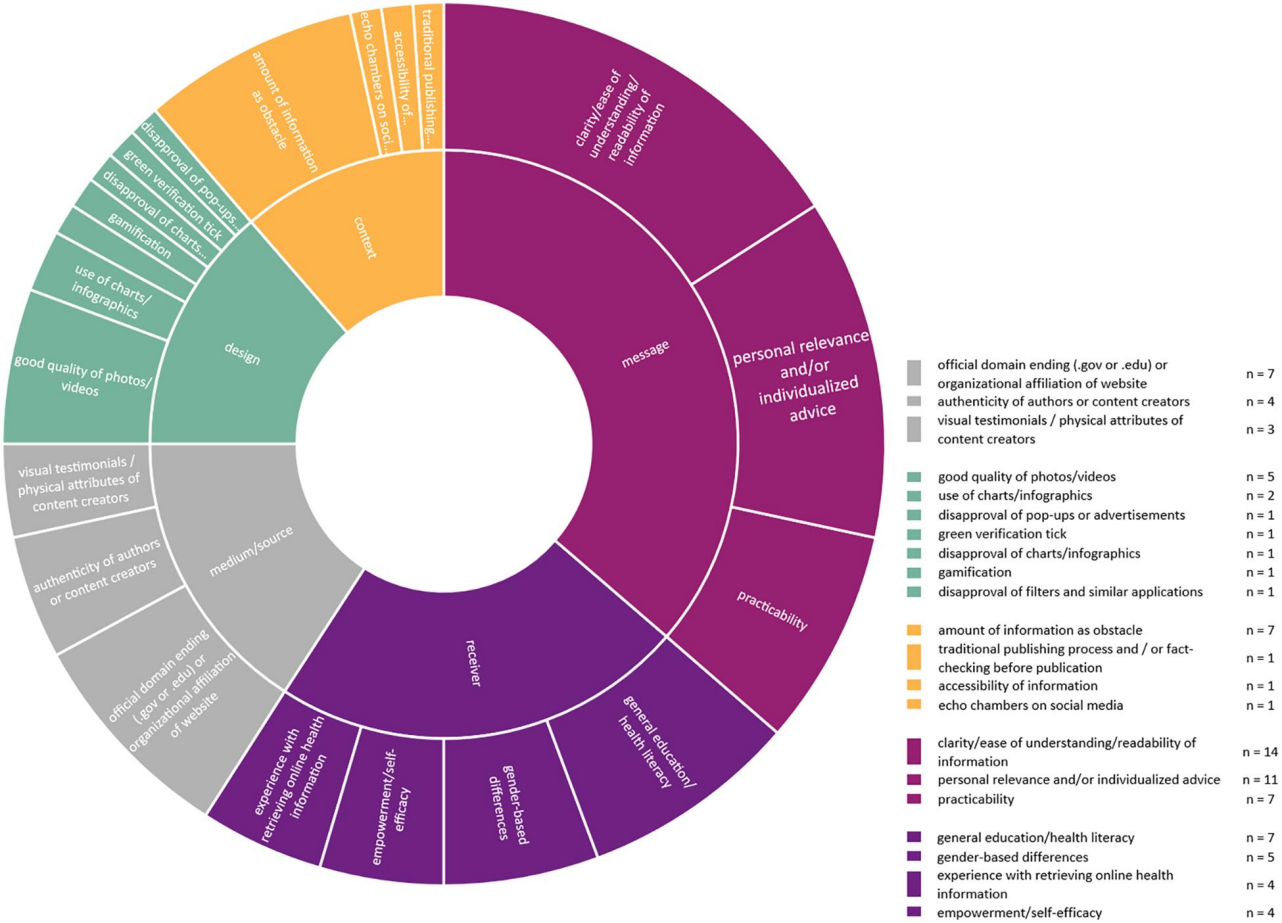
Three most reported new factors per category (full list of factors in Supplementary File 4).

In summary, the two frameworks provided a useful basis for coding the factors influencing the development of trust and credibility, but additional factors need to be considered. Figure 8 provides an overview of all areas and, for each area (receiver, information or message, medium or source, context, and design), shows only the factors reported most frequently, second most frequently, and third most frequently. A comprehensive overview of all identified factors is available in Supplementary File 4.

## 4 Discussion

The scoping review aimed to analyze the digital information sources used by young adults for a healthy diet and the factors that contribute to the formation of trust or credibility in those sources. A total of 286 different reports of information sources were identified across 48 papers. Most of these papers (*n* = 27) cited the internet as a source of nutrition information but provided no additional details. Several papers did not specify the specific platforms, websites, or services that young adults use, trust, or endorse. Given the diverse digital information landscape in which young adults find themselves today, further research should incorporate changes in the media landscape, including diverse platforms, new ways of receiving, sharing, and applying information.

Moreover, it is noteworthy that the list of the most reported information sources, when combined with the search for digital nutrition information, includes three personal sources (healthcare professionals, nutritionists/dietitians, and friends/peers). This suggests that online information is still supplemented by personal encounters, which are characterized by longstanding relationships (such as with friends), trust placed in the person, or acquired expertise in a particular area (such as through targeted appointments with nutritionists/dietitians).

The most frequently reported social media platform is YouTube, appearing in 25% of the papers (*n* = 12). The fact that young adults consult health professionals and nutritionists as often as they consult blogs and social media can be seen as a significant issue, as these platforms lack effective mechanisms for ensuring evidence-based nutrition information. Both types of information sources (blogs and social media) are utilized by different stakeholders, such as traditional, established health organizations with high-quality standards, business owners who try to sell their products, and private individuals who have easy access to publish information on nutrition guidance. For laypeople, it is not always clear to what extent the proposed content is evidence-based. In addition, the algorithms used by social media platforms create so-called “echo chambers”, which are filter bubbles that present the same or very similar information and opinions repeatedly. This is particularly problematic, given that the repetition of information can lead people to trust it (98, 102, 118).

A final statement regarding the most trustworthy sources cannot be made based on the included papers. Many papers do not provide any relevant information on these aspects, and those that do deliver mixed results. 65% of the papers (*n* = 31) do not offer insights into the most trusted source, while 83% (*n* = 40) fail to provide information on the least trusted source. Further the trusted information sources reported in grey and scientific literature differ. Therefore, future research should incorporate both grey literature and scientific articles to obtain the most comprehensive picture possible.

The second research question was: “What factors contribute to young adults considering digital sources of information on healthy diets to be trustworthy or credible?” In total, 82 factors were identified, including 46 that were newly discovered and not covered by any of the frameworks used (Figure 8, Supplementary File 4). The discovery of such a large number of new relevant factors indicates that questions regarding the establishment of trust and credibility of nutrition information have not received enough attention, especially in an environment of an ever-changing information landscape that incorporates new platforms and formats of information, as well as a shift in power dynamics between traditional gatekeepers and new content creators, and a rise in mis- and disinformation.

The newly identified factors encompass all five investigated domains (Supplementary File 4). Across all coded text passages, message-, receiver-, and medium/source-related factors were most frequently reported (*n* = 75, *n* = 69, *n* = 51). This finding suggests that prior research has predominantly focused on these areas, while the other two, context and design, may not have received sufficient attention.

The rise of platforms like Instagram has led to a notable shift in how users perceive and interact with visual elements on the internet and in virtual communication. Other studies indicate that users’ viewing habits change significantly with the duration spent on these platforms (122, 123). Factors such as algorithm-driven content curation and the prevalence of visually compelling imagery play a pivotal role in this transformation. As young adults spend a considerable amount of their daily time on social media, often exceeding several hours, there is a need for in-depth studies on the design elements of digital nutrition information. Further investigation into how design elements can foster trust and credibility could yield important insights in an increasingly visual digital landscape. Research findings from the field of nudging could serve as a starting point, as they have already addressed the design of information, framing, and trustworthiness, as well as ethical issues in this context (124–128).

9 papers had a sample consisting predominantly of females. Thus, our findings may highlight issues that are particularly relevant to women. Gender disparities in both nutrition and health research may play a role in elevated rates of diet-related chronic diseases among men. Other research suggests that when nutrition studies fail to consider male-specific needs and motivations, they risk overlooking important opportunities to improve men's dietary behaviors and overall health (129). Considering that men are often more challenging to engage through traditional, nutrition-focused health promotion strategies (129, 130), further research should investigate nutrition-related topics that specifically appeal to men.

A notable finding is that topics such as ‘healthy diet for athletes’ and ‘dietary supplements’ as part of a healthy diet were addressed in several papers. Five papers address these topics, and two other papers at least inquire about the use of dietary supplements, although they tend to discuss a healthy diet in general terms. Three additional papers describe personalized, optimized nutrition to ensure a healthy diet, a trend likely to increase in the coming years. They also link this trend to athletes and their performance goals. However, some papers also reveal frequent misinformation about dietary supplements and knowledge gaps among consumers of these products, as previously described by other research studies (131–135).

It has to be noted that a significant proportion of the papers analyzed originate from the USA, with study participants enrolled at the university. Given the USA's education and financial system, the likelihood that these study participants were enrolled at the university on athletic scholarships is high. This might constitute a highly selective sample with specific interests due to their living situation. These are circumstances that are not found to that extent in Europe. Thus, the transferability of these findings to European contexts may be limited. Furthermore, it is possible that either no studies on athletes’ online information-seeking behavior exist in Europe, or that our search terms have not captured these studies because supplements are classified differently or framed in distinct ways. This suggests a need for further, more targeted searches and dedicated studies in European settings concerning this topic.

Findings of ethnographic studies suggest that trust is shaped by a complex interplay of national context, cultural values, power dynamics within society, and the integration of minorities (136). Research from other scientific fields, such as the political sciences, supports this view by finding that nationality and ethnicity influence trust and credibility (137).

75% papers did not disclose the ethnicity of their participants (*n* = 36), with ethnicity being reported primarily for countries known for high immigration rates, such as the USA and Canada. To date, health research has primarily focused on general trust in health systems (70) and cross-country comparisons regarding trust in general health information (69). Some studies have explored the interplay of trust and credibility within specific health topics, such as COVID-19 and susceptibility to misinformation (138). Our search did not include any papers that analyzed how aspects such as cultural values, ethnicity, or nationality relate to trust in or the credibility of nutrition information. This finding suggests that research on the interplay between trust, credibility, nutrition, and ethnicity or cultural values remains lacking. This also incorporates regional differences across parts of a country, the perspectives of minority groups, questions of digital (health) literacy among underserved groups, and how they navigate complex online environments. The majority of the included papers cover high-income countries (93.8%), while only three studies cover middle-income countries [*n* = 1 upper-middle-income (South Africa), *n* = 2 lower-middle-income (Ghana & Jordan)].

Some credibility- or trust-inducing factors play nearly equivalent roles for both traditional websites and social media (e.g., the “practicability of the information” and the “amount of information as an obstacle” to building trust). However, for many factors, their influence varies in strength. For example, an individual's “prior background knowledge” is more often reported for evaluating website content. In contrast, factors such as the “authenticity” or “likeability” of the authors are more frequently reported than other factors with regard to evaluating social media content. These findings may be of particular interest to healthcare organizations as they compete with other stakeholders on online platforms for the attention of their target audiences, providing them with evidence-based, trustworthy health information.

Furthermore, across nearly all domains (receiver, information/message, medium/source, context, design) and factors, it is also evident that most factors are described significantly more frequently in relation to website content. This suggests that the dissemination of nutrition information across various social media platforms needs further investigation. In this context, the various formats used to share content (text posts, audio, videos, podcasts, etc.) should be examined, along with how they affect credibility. This seems particularly relevant, given that previous studies have yielded mixed results regarding the extent to which information format influences its credibility (139–141).

### 4.1 Limitations

This study has certain limitations.

While we conducted our search without language restrictions, only studies and grey literature documents in German and English were included from the full-text stage onward. Papers not published in English or German were excluded and marked accordingly to report the number we lost. Further, papers from non-German or non-English-speaking organizations had to be excluded. Only one study from East Asia (i.e., South Korea) was included in the sample. Many East Asian nations are characterized by the strong integration of digital platforms and services into everyday life. Accordingly, it seems likely that information on healthy diets will also be published, searched for, and shared on popular digital platforms in this region (e.g., Weibo or KakaoTalk). This could indicate that either keywords other than “healthy diet” are used there, or that information on healthy diets is mainly published in the respective national languages. Therefore, further research on this topic, incorporating grey literature, should also integrate documents published in the respective national languages.While we followed the agreed-upon and within the protocol published inclusion and exclusion criteria to ensure methodological rigor, the screening of grey literature by a single researcher (CAO) might have introduced potential selection bias.

In addition, we searched for grey literature only on the websites of a few organizations that provide information on healthy eating (Supplementary File 3). This list can be expanded for future studies that may also search in other languages.

While the purpose of a scoping review is to map the characteristics and breadth of available literature for a specific topic, and because the included sources vary in design and methodological quality, no formal critical appraisal was undertaken. This limits the interpretation of the robustness and reliability of the reported findings.

More than half of the papers recruited university students, individuals with degrees, or nutrition experts, while others did not report this. Although training or university degrees are not universally equivalent across countries, individuals with higher levels of education often have better health literacy and greater financial resources. In particular, greater financial resources can enable individuals to prioritize their health (e.g., by spending more on high-quality fruits and vegetables rather than inexpensive, highly processed products). These factors could have influenced the overall findings of this scoping review. Moreover, educated individuals are more likely to engage in preventive health measures and adopt healthier lifestyle choices, which could also have influenced the data and trends identified in the scoping review.

Further research is necessary to account for the diverse educational backgrounds and distinctive perspectives of the general population, as the findings suggest that prior knowledge and education are crucial factors shaping trust and credibility in dietary information. In this context, it is also important to think across disciplines and topics, as the online search for nutrition information overlaps significantly with nutrition literacy and digital health literacy.

Given that digital health literacy is an increasingly relevant topic, future research could focus on the extent to which (near-)global access to digital platforms and current developments in artificial intelligence will change health content relating to healthy diets. This raises the question of whether health content will become increasingly personalized and thus more appealing, or whether it will become increasingly homogeneous due to the international information landscape.

The majority of the included papers cover high-income countries (93.8%). Only three studies cover middle-income countries [*n* = 1 upper-middle-income (South Africa), *n* = 2 lower-middle-income (Ghana & Jordan)]. An important aspect of criticism is the inadequate representation of perspectives from countries with different cultural backgrounds, such as the Arab region, Asia, or Africa, and a substantial absence of representation from low-income countries, even though online information is globally accessible and, in some cases, subject to local access restrictions. This highlights the need for further research that incorporates diverse cultural viewpoints and incorporates the ethnic background of the study participants as an important factor.

### 4.2 Conclusions

It is important to emphasize the need for further research into the credibility and trustworthiness of online nutrition information. The platforms young people frequently use to access information about healthy eating should receive particular attention, as the information landscape has evolved significantly in recent decades. As a consequence, young adults may be exposed to information of variable evidentiary quality. To explore this field more comprehensively, various disciplines must collaborate and share their insights, including information sciences, digital public health, behavioral nutrition, ethnography, health education, and psychology. Additionally, future research should engage diverse target groups, including different cultural communities and minority populations, to guarantee that health and nutrition information is accessible, understandable, and applicable to them. Moreover, the rising prevalence of lifestyle-related diseases underscores the importance of healthy nutrition, as many of these conditions can be prevented through healthier habits.

A greater focus on the design of health and nutrition information, as well as on the contexts in which individuals encounter it, is necessary. This is especially important in light of the rise of AI tools, which will likely increase the prevalence of noise and, therefore, intensify the need to question credibility and trustworthiness.

In terms of interventions, future actions should prioritize equipping young people with the skills to identify trustworthy and credible information effectively. In this regard, it may be beneficial to integrate these initiatives into existing systems, such as schools and universities, as well as other places where teenagers and young adults live, work, and continue their education.

## Data Availability

The original contributions presented in the study are included in the article/Supplementary Material, further inquiries can be directed to the corresponding author.
