# Supplementary material for: Evaluating online nutrition information: a scoping review of young adults’ source preferences and criteria for credibility and trustworthiness
Source: Front Digit Health. 2026 Jun 26;8:1784563. doi: 10.3389/fdgth.2026.1784563 (PMC13350178; doi:10.3389/fdgth.2026.1784563)
Supplement: Supplementary file 2 [file Datasheet2.pdf]

Supplemental File 2:
Search Strategy in MEDLINE via Pubmed

("Trust"[Mesh] OR Trust*[tiab] OR credib*[tiab]) AND ("Young Adult"[Mesh] OR "young adult*"[tiab] OR "student*"[tiab]) AND (information[tiab] OR knowledge[tiab] OR advice[tiab] OR "Consumer Health Information"[Mesh:NoExp] OR "Knowledge"[Mesh]) AND ("healthy diet*"[tiab] OR diet*[tiab] OR fruit*[tiab] OR vegetable*[tiab] OR nut[tiab] OR nuts[tiab] OR snack*[tiab] OR protein*[tiab] OR meal*[tiab] OR plant-based[tiab] OR "whole grains"[tiab] OR vitamin*[tiab] OR "Diet, Healthy"[Mesh] OR "Diet, Plant-Based"[Mesh] OR "Diet"[Mesh] OR "Food"[Mesh] OR "Food, Organic"[Mesh] OR "Functional Food"[Mesh] OR "Dietary Supplements"[Mesh] OR "Nutrients"[Mesh] OR "Meals"[Mesh] OR "Breakfast"[Mesh] OR "Whole Grains"[Mesh] OR nutrition*[tiab] OR vegan*[tiab] OR vegetarian*[tiab] OR breakfast[tiab] OR lunch[tiab] OR dinner[tiab] OR "organic food*" [tiab] OR "functional food*"[tiab] OR fish[tiab] OR seafood[tiab] OR "dietary supplement*"[tiab])

Search date: 10th December 2024

Results: 232
