# Supplementary material for: Evaluating online nutrition information: a scoping review of young adults’ source preferences and criteria for credibility and trustworthiness
Source: Front Digit Health. 2026 Jun 26;8:1784563. doi: 10.3389/fdgth.2026.1784563 (PMC13350178; doi:10.3389/fdgth.2026.1784563)
Supplement: Supplementary file 3 [file Datasheet3.pdf]

Evaluating Online Nutrition Information: A Scoping Review of Young Adults' Source Preferences and Criteria for Credibility and Trustworthiness (C. A. Omane & S. Forberger, 2026)

Supplemental File 3: List of searched health organizations

database	web address	search until (date)	search terms	results
Academy of Nutrition and Dietetics	https://www.eatright.org/	10 december 2024	"healthy diet" "trust" "credibility" "information"	0 results
Europäische Behörde für Lebensmittelsicherheit (EFSA)	https://www.efsa.europa.eu/de	10 december 2024	"healthy diet" "trust" "credibility" "information"	1 result which does not fit the context.
European Commission	https://commission.europa.eu/food-farming-fisheries_de	10 december 2024	"ernährung "vertrauen" "glaubwürdigkeit" "information"	100 pages of results that are not relevant to the topic: occupational safety, health unrelated to nutrition, etc.
European Commission	https://commission.europa.eu/food-farming-fisheries_de	10 december 2024	"healthy diet" "trust" "credibility" "information"	100 pages of results:<br><br>- Results are now almost exclusively related to (healthy) nutrition.<br>- However, they only refer to possible funding opportunities in this area.<br>- The quality of the results declines significantly from page 6 onwards.

FactCheck.org	https://www.factcheck.org/	10 december 2024	"healthy diet"<br>"trust"<br>"credibility"<br>"information"	0 results
Federal Centre for Nutrition<br><br>*Bundeszentrum für Ernährung (BZfE)*	https://www.bzfe.de/info/ueber-das-bzfe/	10 december 2024	"ernährung<br>"vertrauen"<br>"glaubwürdigkeit"<br>"information"	1985 results:<br><br>- Information for the German context only<br>- Back in the search results until 1 January 2020
Food and Agriculture Organization for the United Nations (FAO)	https://www.fao.org/home/en/	10 december 2024	"healthy diet"<br>"trust"<br>"credibility"<br>"information"	13600 results:<br><br>- First 150 results screened: Many results related to food safety and regulations designed to ensure it.
Food Insight	https://foodinsight.org/	10 december 2024	"healthy diet"<br>"trust"<br>"credibility"<br>"information"	37 results:<br><br>Many results that are very relevant to the topic (science communication, Gen Z surveys, trust and credibility)
Global Nutrition Cluster (GNC)	https://www.nutritioncluster.net/	10 december 2024	"healthy diet"<br>"trust"<br>"credibility"<br>"information"	716 results<br><br>Impression after reviewing the first 400 hits:<br>- Many documents on nutrition information systems (surveillance and policy)<br>- Many documents on nutrition during pregnancy and in times of crisis (wars, Covid, etc.)<br>- Much on low- to middle-income countries

International Food Information Council (IFIC)	https://ific.org/	10 december 2024	-	The website does not offer a search function, but it does have a resources section. To access this, you will be redirected to: https://foodinsight.org/
International Obesity Task Force (IOTF)	-	10 december 2024	No search performed as the organization has no own website.	
International Union of Nutritional Sciences (IUNS)	https://iuns.org/	10 december 2024	-	The website does not offer a search function, and most of the links to other sites are outdated and lead nowhere.
Nutrition International	https://www.nutritionintl.org/	10 december 2024	"healthy diet"<br>"trust"<br>"credibility"<br>"information"	0 results
United Nations Educational, Scientific and Cultural Organization (UNESCO)	https://www.unesco.org/en	10 december 2024	"healthy diet"<br>"trust"<br>"credibility"<br>"information"	17136 results<br><br>First 156 results scanned: many results related to trust, information and media literacy, and some also related to science communication. However, none of these results were related to healthy eating and associated sources of information.
World Health Organization (WHO)	https://www.who.int/health-topics/nutrition#tab=tab_1	10 december 2024	"healthy diet"<br>"trust"<br>"credibility"<br>"information"	3 pages with search results mainly relating to: components/definitions of a healthy diet<br>- Individual hits relating to food packaging (labels)

World Obesity Federation (WOF)	https://www.worldobesity.org/	10 december 2024	"healthy diet"<br>"trust"<br>"credibility"<br>"information"	146 results:<br><br>The search returns approximately 20-30 content hits and then just an incredible number of image files. A few links looked promising at first glance, but upon closer inspection, they were unusable.
