# Supplementary material for: Evaluating online nutrition information: a scoping review of young adults’ source preferences and criteria for credibility and trustworthiness
Source: Front Digit Health. 2026 Jun 26;8:1784563. doi: 10.3389/fdgth.2026.1784563 (PMC13350178; doi:10.3389/fdgth.2026.1784563)
Supplement: Supplementary file 4 [file Datasheet4.pdf]

Supplemental File 4:
Trust and credibility influencing factors concerning nutritional health information

Area	Factor	Misinformation Receptivity Framework Reference [1]	Online Credibility Framework Reference [2]	Nutrition-specific (new)
**receiver**	motivation (need for information)	-	"[...] highly involved/motivated users take a central route, relying less on peripheral cues and more on the content of the message) [...]." "User need for information is correlated to credibility judgments—the greater the need, the more likely the user is to accept the information as credible."	-
**receiver**	relevance of the issue	-	"If the user [...] finds the information personally relevant [...], they should persevere beyond the barrier of inconsistent peripheral cues (i.e., bad interface), to the next level of judgment: the information itself."	-
**receiver**	issue involvement	-	"In the determination of credibility, source effects interact with receiver-related factors, such as level of involvement and attitudes (Petty & Cacioppo, 1990). If the audience is less involved with the topic, source characteristics will have more influence on the construct under investigation (e.g., knowledge/attitudes/behaviors)."	-

**receiver**	"social location" of the receiver	-	"Olaisen (1990) discusses factors related to information quality and how "cognitive authority" is established for electronic information. […] He states: "when we are processing information we will give credit and authority to certain persons and sources depending on our social location. One's social location . . . will greatly influence quality factors like credibility (i.e., reliability), relevance and perceived value of information." "	-
**receiver**	consistency with the identity of the receiver	"Identity […] as a causally linked variable that influences beliefs about a given claim (hypothesis) and can shape the interpretation of presented evidence in favor or against the hypothesis."	-	-
**receiver**	consistency with the worldview/value of the receiver	"In cases where the information is consistent with the receiver's worldview, the ultimate interpretation might involve wholesale adoption of the information and stronger convictions in prior beliefs."	-	-
**receiver**	susceptibility for manipulation/misinformation/persuasiveness	"The reliability-weighted inference process underpinning receptivity to political misinformation may thus depend on psychological individual differences and the degree to which one is	-	-

		susceptible to manipulation and interference."		
**receiver**	psychological individual differences	"The reliability-weighted inference process underpinning receptivity to political misinformation may thus depend on psychological individual differences and the degree to which one is susceptible to manipulation and interference."	-	-
**receiver**	prior beliefs	"The individual estimates that the information is highly reliable […] and they have weak prior knowledge or ideological beliefs about the topic, they are likely to accept the misinformation and shift their beliefs in line with the false news."	„Values/beliefs/situation“	-
**receiver**	(ideological) distance between new information and prior beliefs	"The "ideological distance" between the misinformation and prior beliefs, and the direction of that difference, matter for the updating process and the resultant interpretation. When the ideological distance between the incoming information and the prior belief is minimal, the generated interpretation will conform more closely to the incoming information."	„Values/beliefs/situation“	-

**receiver**	prior background knowledge	"The individual estimates that the information is highly reliable […] and they have weak prior knowledge or ideological beliefs about the topic, they are likely to accept the misinformation and shift their beliefs in line with the false news."	„Prior knowledge of the receiver"	-
**receiver**	state of high cognitive load/impaired attential ressources	"A general state of high cognitive load or impaired attentional resources could render individuals more persuadable, gullible, and likely to share false information with others."	„Distraction/noise"<br><br>(Note: Wathen & Burkell originally categorized this factor in the context category. Therefore, this factor can also be found within that category.)	-
**receiver**	general state of stress or relaxation	"Feeling generally stressed or threatened could lead individuals to rely strongly on their prior beliefs and discount incoming evidence, even from reliable sources."	-	-
**receiver**	stereotypes about the source or topic	-	"Users react directly to the qualities of the information source; they also utilize assumptions (e.g., stereotypes about group membership) […] in their assessment of credibility."	-
**receiver**	gender-based differences	-	-	Gender affects how information is sought, received and interpreted.
**receiver**	experience with retrieving online health information/self-confidence	-	-	The acquired experiential knowledge of how to seek health information (and possibly a resulting confidence in the process and/or

				one's own abilities) affects the formation of trust and credibility.
**receiver**	general education/ health literacy	-	-	General education or health literacy functions as a basic prerequisite for the evaluation of information.
**receiver**	differences in information behavior relating to age groups	-	-	The age of individuals determines how they judge sources of information (critical view vs. 'naive' view) and whether information on healthy eating is generally given a high priority.
**receiver**	empowerment/self-efficacy	-	-	The search for information is perceived as empowering or is only taken up in order to (re-)gain control over one's own body.
**receiver**	financial status	-	-	Financial status has an impact on how health information is sought and used. The implementation of information that is associated with (high) costs is avoided.
**receiver**	natural remedies rather than conventional medicine	-	-	Individuals are more likely to look for natural ways (e.g. through diet) to improve their health status instead of consulting a medical doctor and taking conventional medication.
**receiver**	distinction between health status and nutrition	-	-	A distinction is made between health and nutrition. The two are not associated with each other, i.e. nutrition is not perceived as a factor influencing the health status.
**receiver**	previous disease experiences/health status	-	-	Previous experience with illness or a person's current health status

				influences how health information is sought, evaluated and implemented.
**receiver**	positive opinion about digital health tools in general	-	-	The general attitude towards digital health applications influences if information is sought in the digital realm.
**receiver**	diet behaviour	-	-	A person's general nutritional or diet health behavior influences how nutritional information is sought, evaluated and implemented.
**receiver**	place of residence/ geographical location	-	-	A person's place of residence or geographical location influences how nutritional information is sought, evaluated and implemented.

Area	Factor	Misinformation Receptivity Framework Reference [1]	Online Credibility Framework Reference [2]	Nutrition-specific (new)
**info or message**	plausability	„We might expect that the more plausible information is, the more reliable it is estimated to be, and therefore the more weight that information will be given in the inference process, relative to prior beliefs. Indeed, there is robust evidence that plausible misinformation is more likely to be adopted than implausible misinformation."	"Slater and Rouner (1996) postulate that credibility assessments are based on [...] plausibility, and whether it is supported by data or good examples."	-
**info or message**	repetition of information	„[...] because misinformation tends to be viewed as more credible when it is encountered repeatedly."	"These authors conclude that people use the same types of criteria for assessing online information as they use for traditional media—indeed they seek out familiarity in this new environment."	-
**info or message**	topic	-	"Wilson and Sherrell (1993) performed a meta-analysis of empirical studies that measured factors influencing credibility perceptions. Studies that qualified for the analysis measured three types of effects: [...] (2) those due to the message (e.g. the issue being communicated) [...]."	-
**info or message**	internal consistency/logic	„[...] Schmid and and colleagues (2023) found that message features of misinformation, such as the degree to which it includes [...] false	"Obviously, the message itself is critical for information credibility. The message should be internally consistent, and clearly presented."	-

		logic can contribute to more damaging misinformation effects. "		
**info or message**	support by data/examples/experts	„[…] Schmid and and colleagues (2023) found that message features of misinformation, such as the degree to which it includes fake experts […] can contribute to more damaging misinformation effects."	"Slater and Rouner (1996) postulate that credibility assessments are based on […] quality of the message, including its presentation, plausibility, and whether it is supported by data or good examples."	-
**info or message**	framing/expectations	"The brain also computes the integration between prior expectations and incoming inputs when it deals with conceptual claims, such as whether […] a scientific hypothesis is worth rejecting in light of contradictory evidence [...]. "<br><br>"[...] when the communication context is noisy[...] people will be uncertain about the reliability of incoming information, and so prior expectations will weigh more strongly on inferential decisions."	„Framing (loss or gain)"	-
**info or message**	ordering of arguments	-	"This research showed that the way a message is presented (e.g. well- written, -produced, -organized) can influence how the source is perceived."	-
**info or message**	accuracy of information	-	-	Trust and credibility are influenced by the fact if the information is accurately or precisely formulated.
**info or message**	date of publication	-	-	The display of the publication of the information (e.g. on a website)

				influences the formation of credibility/trust.
**info or message**	clarity/ease of understanding/ readability	-	-	The extent to which the information is formulated in an easily understandable manner influences the building of credibility/trust.
**info or message**	comprehensivenes s	-	-	The amount to which the information is complete (this incorporates advantages and disadvantages) influences the trustworthiness or credibility of information.
**info or message**	links to further information citation	-	-	The provision of additional links or citation references enhance the formation of trust or credibility.
**info or message**	absence of bias	-	-	The presentation of information without prejudice facilitates the formation of trust or credibility.
**info or message**	individualized advice	-	-	Personalized or individualized advice influence the development of trust and credibility.
**info or message**	confidentiality	-	-	The information is handled in accordance with data protection regulations. This enhances trust/credibility.
**info or message**	practicability	-	-	Being able to easily implement information into everyday life promotes credibility and trust.
**info or message**	use of positive language/incentive s	-	-	The use of positive or motivating language to convey the information enhances the formation of trust and credibility.

**info or message**	short messages or videos instead of long texts	-	-	The use of short texts, keywords or videos (instead of long texts) promotes the credibility of/trust in information.
**info or message**	sharing of personal experience	-	-	The enrichment of factual information with personal experiences or anecdotes promotes credibility/trust.
**info or message**	use of medical terminology	-	-	The use of medical terminology influences the development of trust/credibility.

Area	Factor	Misinformation Receptivity Framework Reference [1]	Online Credibility Framework Reference [2]	Nutrition-specific (new)
**medium or source**	expertise/knowledge of the source	-	"Expertise/Knowledge"	-
**medium or source**	reputation of the source	"News items that originate from reputable sources tend to be judged as more credible than independent blogs, unknown news sources, or websites with a stated or implied ideological agenda."	"News items that originate from reputable sources tend to be judged as more credible than independent blogs, unknown news sources, or websites with a stated or implied ideological agenda."	-
**medium or source**	popularity of the source	-	"News items that originate from reputable sources tend to be judged as more credible than independent blogs, unknown news sources, or websites with a stated or implied ideological agenda."	-
**medium or source**	credentials	-	"Credentials"	-
**medium or source**	likeability/goodwill of the source	-	"Likeability/Goodwill/Dynamism"	-
**medium or source**	visual testimonials/ physical attributes of content creators	-	-	The information source (e. g., content creator) has physical attributes that show that healthy eating has a positive effect on the outer appearance which, in turn, facilitates the formation of trust/credibility.
**medium or source**	recommendation of source by health professional	-	-	The recommendation of the information source by a health professional enhances trust and credibility.

**medium or source**	official domain ending or organizational affiliation of website/author	-	-	Web domains or authors with connections to the government (e. g. ".gov") or official organizations (e. g. ".org") are deemed as more trustworthy/credible than those who do not have this feature.
**medium or source**	celebrity status	-	-	Popular personalities, such as celebrities, are deemed trustworthy or credible, because they are credited with being familiar with nutrition and a healthy lifestyle.
**medium or source**	disclosure of authors	-	-	A clear naming of the authors of the content facilitates trust or credibility.
**medium or source**	authenticity of authors or content creators	-	-	Authors who show themselves naturally (without e. g. image revision) are deemed trustworthy/credible. This may also include awareness of cultural factors, experiences and matters of representation. Different types of proofs are acceptable.
**medium or source**	selling or product endorsement (negative factor)	-	-	Advertising for products is perceived as an obstacle to building trust/credibility.
**medium or source**	too many followers on social media accounts	-	-	Excessive follower numbers are interpreted as an indication that they may have been bought. This raises concerns about the credibility or trustworthiness of the medium or the source of information.
**medium or source**	verified accounts	-	-	The information originates from a verified account of a health

				professional (i.e. verification similar to twitter etc.) which is interpreted as trustworthy/credible.
**medium or source**	influence of medium or source remains unclear	-	-	The influence of medium or source characteristics on the building of trust or credibility from a recipients' view remains unclear.
**medium or source**	Unclear professional qualification as obstacle	-	-	An unclear professional background of the information's author prevents the formation of trust or credibility.

Area	Factor	Misinformation Receptivity Framework Reference [1]	Online Credibility Framework Reference [2]	Nutrition-specific (new)
**design**	surface attractiveness of digital platform	-	" [...] surface credibility is evident in the assessment of Internet information. Information coupled with a well-designed interface and attractive graphics may result, in the absence of more substantive cues, in a tendency for users to make a positive credibility judgment (Critchfield, 1998). Some surface characteristics, such as errors unrelated to content, negatively influence credibility."	-
**design**	design interface	-	"There is some evidence of an interesting interaction between user characteristics and errors in an electronic information interface. User expertise tends to influence how computing devices and electronic information are perceived [...]."	-
**design**	speed of loading	-	"The authors conclude that there are seven criteria that affect credibility of Web information: [...] and speed of loading."	-
**design**	usability/accessibility	-	"Olaisen (1990) among others [...] differentiates between factors related to sources/messages/receivers and those related to design features of the medium. Calling the former "cognitive" qualities, and the latter "technical" qualities, he ranks various factors from	-

			each in terms of importance [...] On the "technical" side, form, novelty, accessibility, and flexibility are key."	
**design**	interactivity / flexibility	-	"Olaisen (1990) among others [...] differentiates between factors related to sources/messages/receivers and those related to design features of the medium. Calling the former "cognitive" qualities, and the latter "technical" qualities, he ranks various factors from each in terms of importance [...] On the "technical" side, form, novelty, accessibility, and flexibility are key."	-
**design**	good quality of photos/ videos	-	-	Support of the information with the help of (moving) image material that makes a high-quality impression, enhances trustworthiness or credibility.
**design**	green verifaction tick	-	-	A green tick which can be used as a visually supporting symbol for verified and trustworthy/credible health websites.
**design**	disapproval of charts/ infographics	-	-	The use of infographics is viewed negatively with regard to the formation of trust/credibility as they sometimes can not be interpreted by individuals with no prior background knowledge.
**design**	gamification	-	-	Playful elements on websites enhance the understanding and trustworthiness or credibility of information.

**design**	use of charts/ infographics	-	-	The use of infographics is viewed positively concerning the formation of trust/credibility.
**design**	disapproval of pop-ups or advertisements	-	-	Pop-ups or advertising on websites signal that the presented information could be untrustworthy or less credible.
**design**	disapproval of filters and similar applications	-	-	Filters or similar functions that artificially change the appearance of the human information source (e. g. content creator) are a sign to be cautious with judgements regarding trust and credibility.

Area	Factor	Misinformation Receptivity Framework Reference [1]	Online Credibility Framework Reference [2]	Nutrition-specific (new)
**context**	communication context (noisy or safe & clear)	"[…] communication context is noisy – such as when there is a crisis of trust regarding the mixing of truth and falsehoods on the platform on which information is encountered."	„Distraction/noise“	-
**context**	relevance of noise to specific claim	"These contexts could create both skepticisms toward incoming information and doubts about the validity of prior beliefs. The ultimate effect of such a communication context on the resulting interpretations might depend on [...] the relevance of the noise and instability to the specific claims being evaluated."	-	-
**context**	time since contact with message	-	"time since message encountered"	-
**context**	systemic (in-)stability	"[…] individuals could become highly persuadable when their world is highly unstable."	-	-
**context**	amount of information as obstacle	-	-	The overabundance of information leads to confusion for the information's recipient and hinders the development of trust/credibility.

**context**	traditional publishing process/fact-checking before publication	-	-	The traditional publication process and fact-checking ensure that only verified and trustworthy/credible information is published.
**context**	echo chambers on social media	-	-	Echo chambers on social media ensure that users reinforce each other's predefined opinions and serve as a space for the circulation of inaccurate information.
**context**	accessibility of information	-	-	The accessibility of information from public sources serves as a marker for trustworthiness/credibility.

1. Zmigrod L, Burnell R, Hameleers M. The Misinformation Receptivity Framework. European Psychologist. 2023 01/01;28:173-88. doi: https://doi.org/10.1027/1016-9040/a000498.
2. Wathen CN, Burkell J. Believe it or not: Factors influencing credibility on the Web. Journal of the American Society for Information Science and Technology. 2002;53(2):134-44. doi: https://doi.org/10.1002/asi.10016.
