# Supplementary material for: Evaluating online nutrition information: a scoping review of young adults’ source preferences and criteria for credibility and trustworthiness
Source: Front Digit Health. 2026 Jun 26;8:1784563. doi: 10.3389/fdgth.2026.1784563 (PMC13350178; doi:10.3389/fdgth.2026.1784563)
Supplement: Supplementary file 5 [file Datasheet5.pdf]

Supplemental File 5:
Included Papers in the Scoping Review

Scientific studies

Authors	Study location	Income level	Study design/ method	Sample size (n)	Participants' nationality and ethnicity	Participants' gender	Participants health status
Abbey et al. (2017)	USA	high-income	cross-sectional	88	• both not reported	Only males	healthy
Escoffery et al. (2005)	USA	high-income	cross-sectional	743	• american (predominantly caucasian/white sample)<br>• multi-ethnic sample	mixed sample	not reported
Gersten & Sanderson (2024)	USA	high-income	cross-sectional	57	• american (predominantly caucasian/white sample)<br>• multi-ethnic sample	mixed sample (female predominates)	not reported
Ghazzawi et al. (2022)	Jordan	middle-income	cross-sectional	381	• Jordanian<br>• ethnicity not reported	mixed sample	not reported
Hayes et al. (2016)	USA	high-income	cross-sectional	48	• american (predominantly caucasian/white sample)<br>• multi-ethnic sample	mixed sample (female predominates)	not reported
Holland (2017)	Canada	high-income	qualitative study (interviews)	60	• canadian<br>• multi-ethnic sample	not reported	not reported

Holmberg et al. (2010)	Australia	high-income	qualitative study (interviews)	8	• australian<br>• ethnicity not reported	mixed sample	not reported
Kolarić et al. (2022)	Croatia	middle-income	mixed-methods (interviews, survey)	144	• croatian<br>• ethnicity not reported	mixed sample (female predominates)	not reported
Kreft et al. (2023)	South Africa	middle-income	cross-sectional	2318	• south african<br>• ethnicity not reported	mixed sample	mixed sample (healthy and ill participants)
Lun et al. (2012)	Canada	high-income	quantitative study (survey)	440	• canadian<br>• ethnicity not reported	mixed sample	not reported
Matusiewicz et al. (2017)	Germany	high-income	quantitative study (survey)	1028	• german<br>• ethnicity not reported	mixed sample	not reported
Montagni et al. (2018)	France	high-income	qualitative study (interviews)	591	• french<br>• ethnicity not reported	mixed sample	not reported
Mooney et al. (2017)	USA	high-income	mixed-methods (focus groups, interviews, survey)	25	• american (predominantly caucasian/white sample)<br>• multi-ethnic sample (1 Hispanic/Latino individual and 25 white individuals)	mixed sample	diseased (cancer survivors)
Nayak et al. (2024)	Canada	high-income	mixed-methods study (Q methodology)	18	• american<br>• ethnicity not reported	not reported	not reported
Paviˇci´c Žeželj et al. (2018)	Croatia	middle-income	quantitative study (survey)	910	• croatian<br>• ethnicity not reported	mixed sample (female predominates)	not reported
Peša Pavlovi´c et al. (2023)	Croatia	middle-income	mixed-methods (interviews, survey)	144	• croatian<br>• ethnicity not reported	mixed sample (female predominates)	mixed sample (healthy and ill participants)
Quaidoo et al. (2018)	Ghana	middle-income	quantitative study (survey)	192	• ghanaian<br>• multi-ethnic sample	mixed sample	not reported

Rennis et al. (2015)	USA	high-income	qualitative study (focus groups)	14	• american (predominantly hispanic sample)<br>• multi-ethnic sample	mixed sample	not reported
Rozmiarek (2024)	Poland	high-income	qualitative study (interviews)	17	• nationality not reported<br>• ethnicity not reported	mixed sample	not reported
Schwartz & Richardson (2014)	Canada	high-income	quantitative study (survey)	706	• canadian<br>• multi-ethnic sample	mixed sample (female predominates)	mixed sample (healthy and ill participants)
Senkowski & Branscum (2015)	USA	high-income	mixed-methods study (qualitative & quantitative observational data)	30	• american (predominantly caucasian/white sample)<br>• multi-ethnic sample	mixed sample (female predominates)	not reported
Shine et al. (2022)	Australia	high-income	qualitative study (interviews)	10	• multi-national<br>• multi-ethnic sample	only females	not reported
Sung & Choi (2017)	South Korea	high-income	quantitative study (survey)	223	• south korean<br>• ethnicity not reported	only males	not reported
Wang et al. (2020)	Canada	high-income	cross-sectional	1207	• canadian<br>• ethnicity not reported	mixed sample (female predominates)	not reported

Grey literature documents

Authors	location	income level	type of document	sample size	Participants' nationality and ethnicity	Participants' gender	Participants' health status
International Food Information Council Foundation (2011)	USA	high-income	online survey (laypersons)	1000	• US-american<br>• ethnicity not reported	mixed sample	not reported
International Food Information Council Foundation (2013)	USA	high-income	online survey (laypersons)	1006	• US-american<br>• ethnicity not reported	mixed sample	mixed sample (healthy and ill participants)
International Food Information Council Foundation (2016)	USA	high-income	website article about online survey (laypersons)	1003	• US-american<br>• ethnicity not reported	not reported	not reported
International Food Information Council Foundation (2017)	USA	high-income	website article about online survey (laypersons)	1002	• US-american<br>• ethnicity not reported	not reported	mixed sample (healthy and ill participants)
Paipongna (IFIC) (2022)	USA	high-income	website article about online survey (laypersons)	not reported	• US-american<br>• ethnicity not reported	not reported	not reported
Reinhardt Kapsak (IFIC) (2024)	USA	high-income	website article about online survey (laypersons)	not reported	• US-american<br>• ethnicity not reported	not reported	not reported
International Food Information Council Foundation (2024)	USA	high-income	online survey (laypersons)	1000	• US-american<br>• ethnicity not reported	mixed sample	mixed sample (healthy and ill participants)
International Food Information Council Foundation (2024)	USA	high-income	handbook	not reported	• US-american<br>• ethnicity not reported	not reported	not reported

Meyer (IFIC) (2015)	USA	high-income	website article	not reported	• US-american<br>• ethnicity not reported	not reported	not reported
International Food Information Council Foundation (2024)	USA	high-income	online survey (laypersons)	3000	• US-american<br>• ethnicity not reported	mixed sample	mixed sample (healthy and ill participants)
Bundeszentrum für Ernährung (2018)	Germany	high-income	press release	not reported	• nationality not reported<br>• ethnicity not reported	not reported	not reported
Bundeszentrum für Ernährung (2022)	Germany	high-income	website article about online survey (experts)	not reported	• german<br>• ethnicity not reported	not reported	not reported
Bundeszentrum für Ernährung (2023)	Germany	high-income	website article	not reported	• nationality not reported<br>• ethnicity not reported	not reported	not reported
Bundeszentrum für Ernährung (2023)	Germany	high-income	website article	not reported	• nationality not reported<br>• ethnicity not reported	not reported	not reported
Bundeszentrum für Ernährung (2022)	Germany	high-income	website article	not reported	• nationality not reported<br>• ethnicity not reported	not reported	not reported
Bundeszentrum für Ernährung (2023)	Germany	high-income	website article	not reported	• nationality not reported<br>• ethnicity not reported	not reported	not reported
Bundeszentrum für Ernährung (2024)	Germany	high-income	website article	not reported	• nationality not reported<br>• ethnicity not reported	not reported	not reported
Bundeszentrum für Ernährung (2023)	Germany	high-income	website article	not reported	• nationality not reported<br>• ethnicity not reported	not reported	not reported

Bundeszentrum für Ernährung (2024)	Germany	high-income	website article	not reported	• nationality not reported<br>• ethnicity not reported	not reported	not reported
Bundeszentrum für Ernährung (2023)	Germany	high-income	website article about online survey (laypersons)	not reported	• nationality not reported<br>• ethnicity not reported	not reported	not reported
Bundeszentrum für Ernährung (2023)	Germany	high-income	website article about conference	not reported	• nationality not reported<br>• ethnicity not reported	not reported	not reported
Nutrition Hub & Bundeszentrum für Ernährung (2022)	Germany	high-income	online survey (experts)	107	• german<br>• ethnicity not reported	not reported	not reported
Nutrition Hub & Bundeszentrum für Ernährung (2023)	Germany	high-income	online survey (experts)	170	• german<br>• ethnicity not reported	not reported	not reported
Nutrition Hub & Bundeszentrum für Ernährung (2021)	Germany	high-income	online survey (experts)	not reported	• nationality not reported<br>• ethnicity not reported	not reported	not reported
